# Domain exchange at the 3’ end of the gene encoding the fratricide meningococcal two-partner secretion protein A

**DOI:** 10.1186/1471-2164-14-622

**Published:** 2013-09-14

**Authors:** Jesús Arenas, Kim Schipper, Peter van Ulsen, Arie van der Ende, Jan Tommassen

**Affiliations:** 1 Department of Molecular Microbiology, Utrecht University, Padualaan 8, Utrecht 3584 CH, The Netherlands; 2 Department of Medical Microbiology, Center for Infection and Immunity Amsterdam, Academic Medical Center, P.O. Box 22660, Amsterdam 1100 DD, The Netherlands; 3 Department of Molecular Microbiology, Vrije Universiteit, de Boelelaan 1085, Amsterdam 1081 HV, The Netherlands; 4 Netherlands Reference Laboratory for Bacterial Meningitis, Academic Medical Center, Amsterdam, The Netherlands

**Keywords:** Two-partner secretion, *Neisseria meningitidis*, Contact-dependent growth inhibition, Gene conversion, Interstrain competition

## Abstract

**Background:**

Two-partner secretion systems in Gram-negative bacteria consist of an outer membrane protein TpsB that mediates the secretion of a cognate TpsA protein into the extracellular milieu. TpsA proteins have diverse, often virulence-related functions, and some of them inhibit the growth of related bacteria. In *Neisseria meningitidis*, several functions have been attributed to the TpsA proteins. Downstream of the *tpsB* and *tpsA* genes, several shorter *tpsA*-related gene cassettes, called *tpsC*, are located interspersed with intervening open-reading frames (IORFs). It has been suggested that the *tpsC* cassettes may recombine with the *tpsA* gene as a mechanism of antigenic variation. Here, we investigated (i) whether TpsA of *N. meningitidis* also has growth-inhibitory properties, (ii) whether *tpsC* cassettes recombine with the *tpsA* gene, and (iii) what the consequences of such recombination events might be.

**Results:**

We demonstrate that meningococcal TpsA has growth-inhibitory properties and that the IORF located immediately downstream of *tpsA* confers immunity to the producing strain. Although bioinformatics analysis suggests that recombination between *tpsC* cassettes and *tpsA* occurs, detailed analysis of the *tpsA* gene in a large collection of disease isolates of three clonal complexes revealed that the frequency is very low and cannot be a mechanism of antigenic variation. However, recombination affected growth inhibition. *In vitro* experiments revealed that recombination can be mediated through acquirement of *tpsC* cassettes from the environment and it identified the regions involved in the recombination.

**Conclusions:**

Meningococcal TpsA has growth-inhibitory properties. Recombination between *tpsA* and *tpsC* cassettes occurs in vivo but is rare and has consequences for growth inhibition. A recombination model is proposed and we propose that the main goal of recombination is the collection of new IORFs for protection against a variety of TpsA proteins.

## Background

*Neisseria meningitidis* is a Gram-negative bacterium that colonizes the human respiratory tract and occasionally causes meningitis and/or sepsis. Disease-related strains can express either one of six different capsular polysaccharides (A, B, C, Y, W_135_ and X). Polysaccharide-based vaccines have been developed against four of these capsule types. Unfortunately, the serogroup B capsular polysaccharide cannot be used as a vaccine due to its poor immunogenicity, while non-capsular antigens seem to be effective only temporarily and in specific geographic areas due to high antigenic variability in meningococci.

Various outer-membrane components and secreted proteins exhibit variable expression and/or antigenic diversity, which plays an important role in immune escape and has serious implications for the development of effective vaccines [1]. The high variability of these antigens is based on different mechanisms, including gene conversion and slipped-strand mispairing [2].

Gene conversion is the non-reciprocal exchange of DNA fragments situated at different chromosomal locations. It is based upon homologous recombination between the donor and recipient loci [3,4]. Virulence-associated surface structures in bacterial pathogens are often subject to antigenic variation by gene conversion. This has been studied extensively for the pilin locus of *Neisseria* spp. [5-7]. The retractile type IV pili of *Neisseria* are involved in attachment to host tissues [8,9], DNA acquisition [10,11] and twitching motility [12]. In addition to the pilin expression locus *pilE*, the genome of these bacteria contains a repertoire of promoter-less *pilS* cassettes, with homologous and variable regions relative to *pilE*. These *pilS* cassettes can unidirectionally be transferred to the *pilE* expression locus, resulting in partial alteration of the pilin sequence. Alternatively, this recombination event can switch off the formation of pili by introduction of a premature stop codon in *pilE*. The frequency of gene conversion seems to be strain and species dependent and can be as high as 0.13 recombination events per cell in the case of gonococcal strain FA1090 [13].

**Figure 1:**
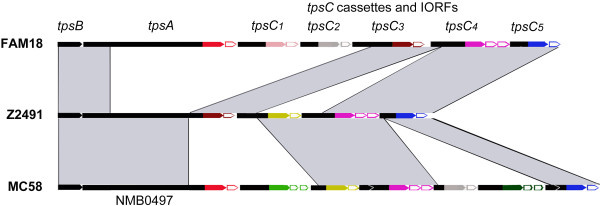
**Comparison of the genetic organization of three TPS islands in different meningococcal strains.** Each island consists from the 5’ to the 3’ end of a *tpsB*, a *tpsA*, and a variable number of *tpsC* cassettes interspersed with intervening ORFs (IORFs) (open arrows). The *tpsA* genes and *tpsC* cassettes can display very different domains of ~450 bp at their 3’ end as indicated by the different colors. Domains with very high sequence similarity are indicated with the same color. Also the IORFs show high heterogeneity and IORFs with high sequence similarity are indicated with the same color. Note that *tpsA* or *tpsC* genes with similar sequences at their 3’ end are always followed by IORFs with high sequence similarity. Regions with high sequence similarity between the different islands are indicated by grey shading, indicating gross rearrangements in the organization between the islands. Note that strain MC58 contains five *tpsA* genes, including two *tpsA* genes of system 1 (Figure S1 in Additional file 1). The island depicted here contains the *tpsA1b* gene with locus tag NMB0497.

In two-partner secretion (TPS) systems, a large protein, generically called TpsA, is secreted through a specific outer membrane transporter, designated TpsB [14,15]. TpsA is synthesized as a precursor with a signal sequence and transported to the periplasm via the Sec machinery. In the periplasm, it interacts via its N-terminally located TPS domain with TpsB and is transported to the cell surface. TPS systems of *N. meningitidis* seem of relevance to pathogenicity as suggested by reported roles in adhesion [16], intracellular survival [17] and biofilm formation [18]. Cluster analysis of the sequences of the TpsB proteins and of the TPS domains of the TpsA proteins revealed the presence of three different TPS systems in *N. meningitidis*[19]. Different isolates of *N. meningitidis* can contain one to three different TPS systems, and some of these systems can contain two *tpsA* genes [19]. In the genome sequence of strain MC58, for example, five different *tpsA* genes were identified, two of system 1 (designated *tpsA1a* and *tpsA1b*), two of system 2 (*tpsA2a* and *tpsA2b*), and one of system 3 (*tpsA3*). In contrast, the genome sequences of strains FAM18, 053442 and Z2491 contain only one single *tpsA*, *i.e.* a *tpsA* of system 1 (*tpsA1* a.k.a. *hrpA*) [19-21], which appears most widespread among meningococcal isolates [19]. The *tpsB* and *tpsA* genes are situated on specific genetic islands on the chromosome. Downstream of the *tpsA* genes, several *tpsA*-related open reading frames (ORFs) are located, generically referred to as *tpsC* cassettes [21], interspersed with small intervening ORFs (IORFs) (see Figure 1 for examples). As compared to *tpsA*, all *tpsC* cassettes are missing extensive portions at the 5’ end including the segment encoding the signal sequence and the TPS domain. They do share sequence similarity with a central part of *tpsA* but show an entirely different 3’ terminal sequence [21]. From this observation, it was postulated that these *tpsC* cassettes could substitute the 3’ end of *tpsA* by gene conversion, thereby causing antigenic variation of TpsA, similarly as in the *pilE/S* system [2,21].

Recently, Aoki *et al*. described that certain TpsA proteins of *E. coli* inhibit the growth of related bacteria [22,23]. This activity is mediated by direct contact between bacteria of the same species through the interaction of TpsA with the conserved outer membrane protein BamA in the target cells [24]. The activity, referred to as contact-dependent growth inhibition (CDI), was also observed in other bacteria [22,25].The CDI activity resides in the C-terminal region of TpsA, which is cleaved off from TpsA after interaction with the receptor and delivered in the cytoplasm of target cells, where it exerts DNase or tRNase activity. Immunity in the producing cells is provided by a small protein encoded by the ORF immediately downstream of *tpsA*. This immunity protein binds the toxic domain of TpsA and inhibits its activity [22]. Like in the *tps* gene clusters of *N. meningitidis*, these *tpsA* genes are often followed by several 5’-truncated *tpsA* homologs displaying entirely different 3’ ends [26]. It was proposed that these *tpsC*s comprise a reservoir of alternative C-terminal regions and that exchange of the 3’ end of *tpsA* by a *tpsC* cassette alters the CDI mechanism [26]. However, so far, no study addressed whether such substitutions at the 3’ end of *tpsA* actually take place.

In the present study, we first investigated whether also the meningococcal TpsA proteins mediate growth inhibition. Then, we investigated whether recombination at the 3’ end of *tpsA* indeed occurs in vivo and what the frequency, the mechanism, and the function of such recombination might be.

## Results

### Meningococcal TpsA1 mediates growth inhibition

To investigate whether meningococcal TpsA proteins may possess growth-inhibitory activity, we initially focused on strain FAM18, which contains a single TPS island with one *tpsA1* gene and five *tpsC* cassettes (Figure 1) [2,20]. However, since this strain appeared non-transformable, we switched to strain B16B6, another reference strain of the same clonal complex (cc), i.e. cc11. Sequencing of the complete TPS island of strain B16B6 (Genbank: HQ420265) revealed exactly the same genetic organization and > 99% sequence identity at the nucleotide level with that of FAM18. The TpsA protein of B16B6 shows only three amino-acid substitutions relative to that of FAM18.

**Figure 2:**
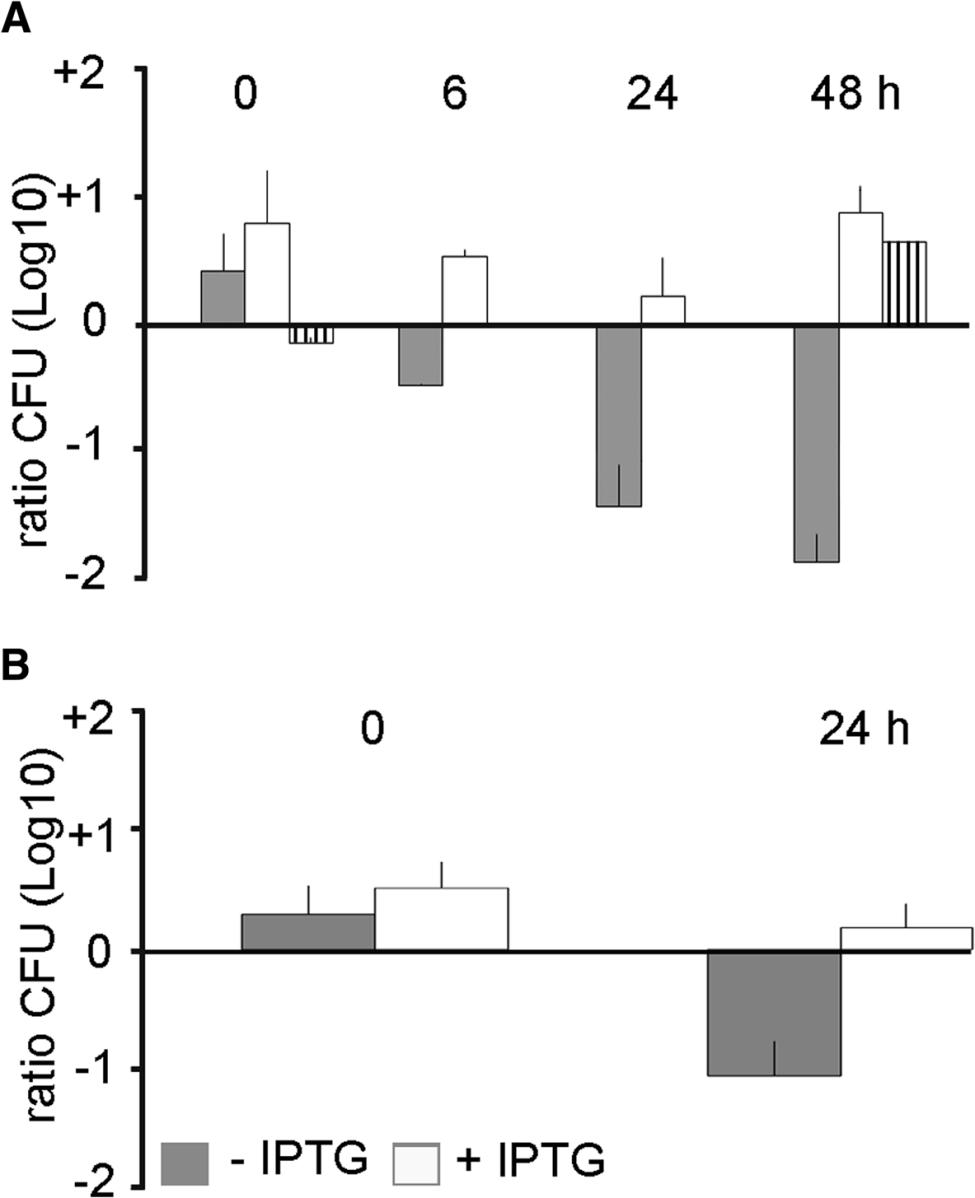
**Growth-inhibitory function of meningococcal TpsA. (A)** Cells of an unencapsulated derivative of strain B16B6, carrying a plasmid with a chloramphenicol-resistance marker, were mixed 1:1 with cells of Δ*tpsA-tpsC*, Δ*tpsB* or Δ*tpsC*_*2-5*_ mutants, all carrying a *kan* cassette. The suspensions were spotted on GC plates without antibiotics and incubated for various time periods. In the experiment with the Δ*tpsC*_*2-5*_ mutant as the target cells, the suspensions were only spotted after 0 and 48 hours. The ratios of the Δ*tpsA-tpsC* (grey bars), Δ*tpsB* (white bars), or Δ*tpsC*_*2-5*_ (hatched bars) mutants over wild-type bacteria in the spots was determined by plating on GC media containing kanamycin or chloramphenicol and counting colony-forming units after overnight incubation. **(B)** A rifampicin-resistant derivative of the unencapsulated B16B6 strain was mixed 1:1 with the Δ*tpsA-tpsC* mutant harboring plasmid pFPIORF_1_, which contains the first IORF of B16B6 under the control of an IPTG-inducible promoter. The bacteria were incubated in the presence or absence of IPTG for 24 hours as above. The ratio of the Δ*tpsA-tpsC* mutant over the wild-type bacteria was determined as described. Results are means and s.d. of three independent experiments. All the strains tested here did not show differences in viability when grown separately.

Next, we constructed a Δ*tpsA*-*tpsC* mutant of strain B16B6 lacking *tpsA* and all *tpsC* cassettes and IORFs. In addition, since the presence of a capsule inhibits CDI in *E. coli*[22], we used capsule-deficient derivatives in all assays described below. When we co-incubated the wild type and the Δ*tpsA*-*tpsC* mutant in liquid medium as described for *E. coli*[23], no growth inhibition of the mutant was observed independent of the media used (TSB, LB, or RPMI), co-incubation time (3–6 hours) or initial ratio of target and killer cells (ranging from 1:1 to 1:1000). It has been reported that meningococcal TpsA affects later stages of biofilm development (48 hours old biofilms) and that its production is increased under anaerobic conditions [18]. Thus, we co-incubated the wild type and the mutant on agar plates in a candle jar with low oxygen concentrations. Under these conditions, growth inhibition of the mutant was observed as a decline in time of the ratio of the colony-forming units (CFU) of the mutant over that of the wild type (Figure 2A, grey bars). In contrast, growth of a Δ*tpsB* mutant, which is defective in the secretion of TpsA but still contains all IORFs, was not affected when co-incubated with the wild type (Figure 2A, white bars). Also, growth of a Δ*tpsC*_*2-5*_ mutant, which lacks *tpsC*_2_ to *tpsC*_6_ and the IORFs in between, but still contains IORF_1_ and IORF_2_, was not affected when co-incubated with the wild type (Figure 2A, hatched bars), suggesting that protection against the growth-inhibiting properties of TpsA is mediated by IORF_1_ and/or IORF_2_.

To confirm the role of the IORF_1_ immediately downstream of *tpsA* in B16B6 in conferring immunity, the corresponding gene was cloned behind an isopropyl-β-D-1-thiogalactopyranoside (IPTG)-inducible promoter on plasmid and introduced into the Δ*tpsA*-*tpsC* mutant. Growth of the complemented strain was inhibited when co-incubated with the wild-type strain in the absence but not in the presence of IPTG (Figure 2B). Thus, the IORF indeed confers immunity to growth inhibition mediated by TpsA. In conclusion, the meningococcal TpsA protein has growth-inhibitory activity and the IORF immediately downstream of *tpsA* provides immunity to this activity.

### Structural organization of meningococcal TpsA1 and TpsC proteins

To study possible genetic exchange between *tpsA* genes and *tpsC* cassettes, we first extended previous bioinformatic analyses using available genome sequences [19,21], thereby focusing on TPS system 1 because of its widespread presence in meningococcal isolates. The genetic organization of TPS system 1 islands in the available genome sequences of strains α14 [27], α153 [27], α275 [27], 053442 [28], 8013 [29], FAM18 [2], MC58 [30] and Z2491 [31] is depicted in Figure S1 in Additional file 1 with selected examples shown in Figure 1.

Multiple sequence alignments of the corresponding TpsA proteins (data not shown) revealed that they are modular and consist of three regions: a highly conserved N-terminal region, a semi-variable central region, and a hyper-variable C-terminal region. The N-terminal region of ~500 amino acids shows > 98% identity between all aligned sequences and contains the TPS domain required for interaction with the TpsB protein in the outer membrane. For the central region of ~900 amino-acid residues, two main allelic variants were identified designated core 1 and core 2 (Figure S2A in Additional file 1). These sequences showed > 19% divergence between the groups and < 2% divergence within the groups. The C-terminal regions of ~600 amino-acid residues, where the toxic activity of TpsA resides, are highly variable with sequence divergence ranging from 5-50%. Sequence divergence is particularly high in the last ~150 residues, where five different groups of sequences can be discriminated among the 10 TpsA proteins compared (Figure S2B in Additional file 1). These C-terminal domains show very high sequence conservation within the groups (> 98%) and extremely low, if any, sequence similarity between the groups.

The polypeptides putatively encoded by the *tpsC* cassettes are variable in length (260–893 residues). In their N-terminal part upstream of a conserved VENN motif (22), they share sequence similarity with TpsA proteins (see Figure S3 in Additional file 1, which shows an alignment of the C-terminal region of TpsA and the putative TpsC proteins of strain FAM18). The corresponding DNA sequences are presumably used for homologous recombination into the *tpsA* locus. Downstream of the VENN motif and particularly within the C-terminal moieties of ~150 amino-acid residues, the sequences are again hyper-variable (Figure S3 in Additional file 1), some sharing high sequence similarity with C-terminal segments of TpsA proteins and others constituting new groups of C-terminal domains. Sequence comparisons identified 11 different groups of TpsA/TpsC C-terminal sequences (indicated by different colors in Figure 1 and Figure S1 in Additional file 1) with > 90% of similarity within the groups (Additional file 1: Table S1). The results of such sequence analyses are consistent with the occurrence in vivo of the suggested gene conversion at the 3’ end of the *tpsA* gene [21]. For example, the *tpsA* of strain Z2491 could have been generated by recombination of a *tpsC* cassette similar to *tpsC3* of strain FAM18 into a *tpsA* like NMB0497 of MC58 (Figure 1). However, it should be noted that also recombination mechanisms other than gene conversion could be responsible for the observed variation in the 3’ end of *tpsA*.

The polypeptides encoded by the IORFs located in between *tpsA* and *tpsC*s (open arrows in Additional file 1: Figure 1 and Figure S1) putatively encode immunity proteins to confer protection against the toxic activity of the products of the directly upstream located *tpsA* or *tpsC*. They can be grouped with > 95% sequence similarity within the groups and very low, if any, similarity between the groups (data not shown). Interestingly, *tpsA*s and *tpsC*s with a similar C-terminal sequence are always immediately followed by an IORF from the same sequence group. IORFs of the same group are colored identically in Figure 1 and Figure S1 in Additional file 1, and, to reflect the interconnectivity with the upstream TpsA/TpsC, the same color is used for the IORFs as for the C-terminal ends of the upstream TpsA or TpsC.

### Replacement of the 3’ end of *tpsA* is rare in cc11 and cc8 strains

The bioinformatic analysis above is consistent with the occurrence of the suggested gene conversion at the 3’ end of meningococcal *tpsA*. However, the TPS islands in the strains analyzed could also have been acquired independently by horizontal gene transfer from different sources. To determine whether recombination of *tpsC* cassettes with the 3’ end of *tpsA* indeed occurs in vivo, we decided to analyze the TPS system 1 of various isolates from the same cc. If recombination indeed takes place at frequencies comparable, for example, to that reported for gene conversion at the *pilE* locus, different *tpsC* cassettes would be expected to be present at the C terminus of TpsA in such isolates. For this purpose, we first focused on cc11, a lineage predominantly associated with invasive disease [32] and therefore useful to evaluate the possible implications of such recombination events during human infection.

The observation that the organization of the TPS island of B16B6 is very similar to that of FAM18 (see above) in spite of the very different isolation years of these strains being 23 years apart (Additional file 2) already suggested that the *tps* loci might actually be rather stable. We then decided to analyze a large panel of 277 cc11 disease isolates collected in The Netherlands between 1960 and 2008 (Additional file 2). We used PCR to assess for possible replacements of the 3’ end of *tpsA* by *tpsC* cassettes as outlined in detail in Figure S4 in Additional file 1; the results for individual isolates are listed in Additional file 2: Table S2. With only three exceptions, which are described in detail in the next section, all isolates showed the presence of the same cassette at the 3’ end of *tpsA* as in FAM18. These results demonstrate that the *tpsA* locus in cc11 strains is remarkably stable. A possible explanation for the observed stability of *tpsA* is that the cassette present at the C terminus of TpsA in cc11 isolates is maintained because it is required for the highly invasive phenotype of these strains or because of reduced interbacterial competition during infection. To investigate this possibility, we also analyzed six cc11 carrier isolates using similar procedures (Figure S4 in Additional file 1), which revealed again the same organization as that in FAM18, suggesting that the 3’ end of *tpsA* is also stably maintained in these isolates and that the lack of exchange is not favored by host invasion.

For comparison, we determined the frequency of another well-known mechanism of genetic variation, *i.e.* phase-variation by slipped-strand mispairing at direct DNA repeats, in cc11 strains. Thus, the expression of the autotransporter NalP and the outer membrane Opa proteins, which both exhibit phase-variable expression [33,34], was evaluated by Western blotting in a panel of 56 cc11 isolates, including both carrier and disease isolates (Additional file 2: Table S2). Of these isolates, 32 were positive for NalP expression (57%) and 23 for Opa expression (41%). Thus, the frequency of recombination at the 3’ end of the *tpsA* gene is clearly much lower than that of another genetic mechanism of variation used by this pathogen during infection.

Finally, we analyzed strains from another hyperinvasive cc, *i.e.* cc8, using the same strategy. The TPS island of cc8 strain NMB was recently reported to have a similar organization as that in FAM18 [18]. We sequenced *tpsA* of another cc8 strain, strain 2996 (Genbank: HQ420264), and found 99% identity at the nucleotide level with *tpsA* of FAM18. PCR analysis targeted for various fragments in the downstream region (Figure S4 in Additional file 1) indicated the same organization of *tpsC*s as in FAM18. Next, a panel of 91 cc8 disease isolates was analyzed (Figure S4 in Additional file 1); all of them appeared to contain the same cassette at the 3’ end of *tpsA* as in FAM18. Thus, recombination at the 3’ end of the *tpsA* locus did not occur in the isolates examined and is therefore rare, also in cc8 strains.

### Characterization of the TPS island in the deviating cc11 isolates

**Figure 3:**
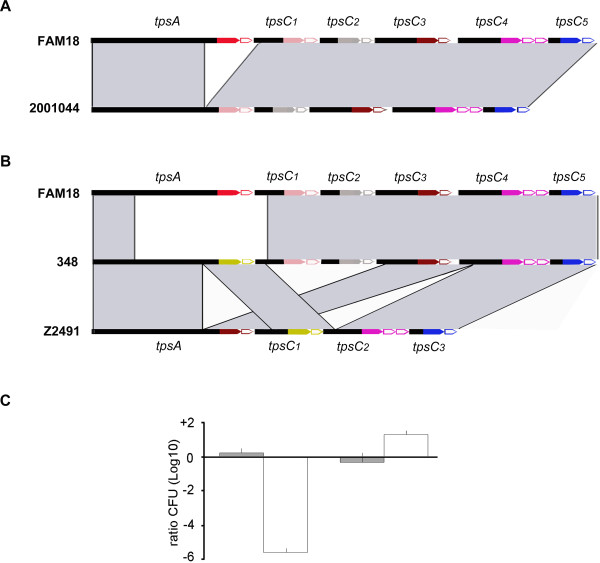
**Genetic organization of the deviant TPS organization in cc11 isolates 2001044 and 348 and consequences in growth-inhibition activity. (A)** Comparison of the TPS organization in cc11 reference strain FAM18 and the clinical isolate 2001044. **(B)** Comparison of the genetic organization of the TPS island in clinical isolate 348 and those in cc11 reference strain FAM18 and cc4 reference strain Z2491. In both panels, regions of high sequence similarity between the islands are indicated by grey shading. Note that the TPS organization in isolate 2001044 could have been generated by intragenomic recombination, whilst that in isolate 348 must have implicated horizontal gene transfer. **(C)** Unencapsulated derivatives of strains B16B6 and its Δ*tpsA-tpsC* mutant, both carrying an erythromycin-resistance marker, were mixed 1:1 with an unencapsulated derivative of strain 2001044 carrying a chloramphenicol-resistance marker, spotted on GC plates and incubated as described in the legend to Figure 2. The ratios of 2001044 bacteria over B16B6 (left) or B16B6Δ*tpsA-tpsC* mutant (right) bacteria were determined after 0 hours (gray bars) or 24 hours (white bars) of incubation by plating on GC media containing erythromycin or chloramphenicol.

The three cc11 isolates that, according to the PCR analysis, deviated from FAM18 at the 3’ end of *tpsA*, *i.e.* isolates 2001044, 2020041 and 348 (Additional file 2), all produced a TpsA as evidenced by Western blotting (data not shown). Further PCR analysis of the TPS island of isolates 2001044 and 2020041 (Figure S4 in additional file 1) indicated that the *tpsC1* cassette had recombined into the *tpsA* locus with loss of the intervening DNA as depicted in Figure 3A. Sequence analysis of the entire TPS island of isolate 2001044 (Genbank: HQ420262) confirmed this suggestion. Of note, *tpsC1* of FAM18 shows 99% sequence identity between bp 195–1628 with a segment of *tpsA* (bp 4218–5651) (Figure S5A in Additional file 1). Comparison of the upstream and downstream sequences indicated that the recombination resulting in the *tpsA* of isolate 2001044 had occurred in this shared region. The sequence downstream of *tpsA* in isolate 2001044 showed > 99% identity with the region downstream of *tpsC1* in FAM18 (Figure 3A). This region was also similar in isolate 2020041 (data not shown). Thus, the results for these isolates demonstrate that replacement of the 3’ end of *tpsA* by *tpsC* cassettes indeed occurs in vivo, albeit at a very low frequency.

Sequence analysis of the complete TPS island of the third deviant cc11 strain, isolate 348 (Genbank: HQ420263), revealed more complex rearrangements (Figure 3B). The encoded TpsA has a central core 2 region like in strain Z2491 instead of a core 1 as in FAM18 (Figure S2A in Additional file 1), and it contains a C terminal end identical to that of TpsC1 of Z2491 (Figure 3B). Thus, compared to Z2491, the 3’ end of *tpsA* is replaced by *tpsC1* in isolate 348. Sequence comparisons (Figure S5B) suggest that the recombination event took place between bp 4174–4605 of *tpsA* and bp 910–1341 of *tpsC1*, where these sequences show high sequence identity.

The IORF downstream of *tpsA* in isolate 348 is identical to that downstream of *tpsC1* of Z2491 (Figure 3B). The subsequent *tpsC1* in 348 showed 94% identity within the first 858 bp with the corresponding 5’ fragment of *tpsC2* of Z2491 (Figure 3B and Figure S5C in Additional file 1). The rest of this *tpsC1* was identical to the 3’ end of *tpsC1* of FAM18 (Figure 3B and Figure S5C in Additional file 1), differing only in one single bp. At the boundary, an 18-bp sequence is shared between the three genes (Figure S5D in Additional file 1), which was likely involved in the recombination event; apparently, only short stretches of 100% identity are required for this. The remaining part of the TPS island of isolate 348 showed > 99% identity with the corresponding region of FAM18 (Figure 3B). Thus, it appears that the *tpsA* of isolate 348 was generated by recombination between a *tpsA* and a *tpsC1*similar to those of Z2491. This *tpsA* gene was then introduced in the cc11 strain by recombination at the 5’ end in the DNA encoding the TPS domain of *tpsA* or in *tpsB* and at the 3’ end within *tpsC1*.

A possible explanation for the low frequency of recombination observed at the 3’ end of *tpsA* in vivo is that such recombination results in the concomitant loss of the downstream IORF, which provides immunity to the TpsA of the parental strain. Thus, within a microbial community such as a biofilm or a microcolony, the growth of a recombinant would be inhibited by its congeners. To investigate whether recombination indeed confers a growth disadvantage in the presence of a wild-type strain, we performed growth-inhibition experiments with the recombinant isolate 2001044 and strain B16B6. The growth of the deviating isolate was drastically reduced when co-cultured with B16B6 (Figure 3C, left bars), presumably because it is not protected against TpsA of B16B6. The growth of strain 2001044 was not inhibited when the strain was co-cultured with the Δ*tpsA-tpsC* mutant of B16B6, which does not produce TpsA (Figure 3C, right bars), demonstrating that the TpsA of strain B16B6 is responsible for the growth inhibition of isolate 2001044. In the latter experiment (Figure 3C, right bars), rather the growth of the Δ*tpsA-tpsC* mutant appeared to be inhibited, indicating that the recombinant TpsA produced by isolate 2001044 has growth-inhibitory activity against which the Δ*tpsA-tpsC* mutant of B16B6 is not protected presumably because it lacks all immunity proteins including that encoded by the IORF immediately downstream of the *tpsC1* cassette. Consistent with this notion is the observation that the growth of the Δ*tpsB* mutant of B16B6, which does not secrete TpsA but has retained all IORFs, was not inhibited by isolate 2001044 (data not shown). To further confirm that the growth of isolate 2001044 is inhibited in the presence of B16B6 because it lacks the IORF immediately downstream of *tpsA* of B16B6 (Figure 3A), the strain was transformed with plasmid pFPIORF_1_, which contains this IORF under *lac* promoter control, and competition experiments with B16B6 were performed in the presence or absence of IPTG. The growth inhibition of the plasmid-containing 2001044 target cells was reduced by 3.1 log units (mean of three independent experiments) in the presence as compared to the absence of IPTG. Together, these data support the hypothesis that recombination at the *tpsA* locus confers a selective disadvantage within a genetically similar community because of the loss of the relevant immunity protein.

### Comparison of the frequency of exchange at the *pilE* and *tpsA* loci

The results described above confirmed that the 3’ end of *tpsA* can be replaced by *tpsC* cassettes in vivo but at a much lower frequency than anticipated considering the reported high frequency of recombination at the *pilE* locus. However, it was reported recently that also gene conversion at the *pilE* locus is not detectable in cc8 and cc11 isolates [35,36]. Although this can be explained by the nature of the *pilE* and *pilS* genes present in these strains (*vide infra*), we cannot exclude the possibility that cc8 and cc11 strains contain some additional genetic defect that reduces the recombination frequency also at the *tpsA* locus. To rule out this possibility, we analyzed another clonal lineage, which is known to be proficient in recombination at the *pilE* locus, *i.e.* cc32 [35], even though this analysis is more complicated by the presence of two *tpsA1* copies in strains of this cc, such as MC58 (Additional file 1: Figure S1).

We examined 50 cc32 disease isolates. First, the region corresponding to the *pilE* locus was amplified by PCR as described [35]. Three isolates did not yield an amplicon, suggestive of loss of the *pilE* locus, whilst two yielded a smaller amplicon than expected suggesting recombination with a *pilS* (Additional file 2). In a subsequent PCR, one of the primers annealed to a variable region in *pilE*. Of the 47 isolates positive for *pilE*, 13 did not yield an amplicon in this PCR (Additional file 2), demonstrating recombination relative to the *pilE* sequence of MC58 in at least 28% of the strains.

Next, recombination at the *tpsA1* loci was examined by PCR as outlined in detail in Additional file 1: Figure S6. This analysis revealed that the *tpsA1b* locus corresponding to NMB0497 of strain MC58 was stably maintained in all isolates. However, the *tpsA1a* locus corresponding to NMB1779 was detected in the initial PCR in only 37/50 isolates suggesting that either recombination had occurred at this *tpsA1* locus in the remaining 13 isolates or the *tpsA1* locus was not duplicated in these isolates. Subsequent PCRs targeting the boundaries of the TPS islands (Additional file 1: Figure S6) revealed the absence of the duplication in 11 of these isolates. In the remaining two isolates, the duplication was detected and thus, apparently, recombination had occurred at the *tpsA1a* locus. Subsequent PCRs (outlined in Figure S6 in Additional file 1) revealed the presence in one of these isolates of a *tpsA1* similar to that of FAM18, *i.e.* with a central core different from that of the *tpsA1* genes of MC58. Therefore, this *tpsA1* must have been acquired in this isolate by horizontal gene transfer. In the other isolate, we detected that the *tpsC5* located downstream of *tpsA1b* had recombined into the *tpsA1a* locus (Figure S6 in Additional file 1). Thus, in 50 isolates containing in total 89 *tpsA1* genes, only a single case of *tpsA*/*C* exchange was detected (*i.e.* 1.1%), in agreement with the low frequency detected in cc11 and cc8 isolates and much lower than the frequency of gene conversion at the *pilE* locus in the same set of strains. Consistent with our results, also in the newly available genome sequence of cc32 isolate H44/76 [37], the organization of the *tpsA1*-containing islands is similar to that in MC58 whilst the *pilE* gene is different.

### Recombination within TPS sequences under laboratory conditions

**Figure 4:**
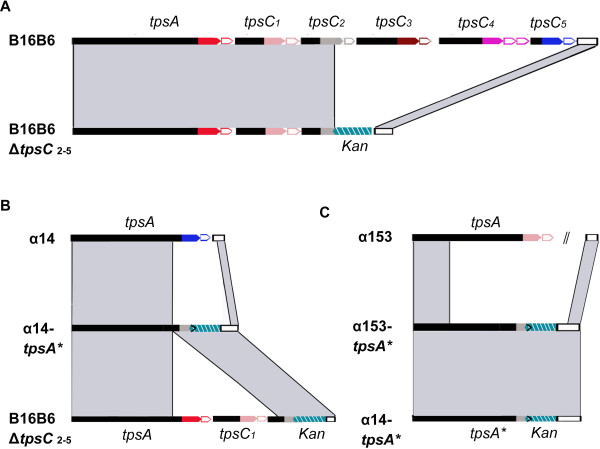
**In vitro recombination in the TPS islands. (A)** Organization of the TPS islands in strain B16B6 and its mutant derivative B16B6Δ*tpsC*2-5 where a *kan* cassette replaces several *tpsC* cassettes and IORFs. **(B)** Organization of the TPS island in recombinant strain α14-tpsA* (middle) obtained after transformation of strain α14 (top) with chromosomal DNA from strain B16B6Δ*tpsC*2-5 (bottom). **(C)** Organization of the TPS island in recombinant strain α153-tpsA* (middle) obtained after transformation of strain α153 (top) with chromosomal DNA from strain α14-tpsA*#1 (bottom). The organization of the TPS island of strain α153 is not completely depicted because it is not located on a single contig in the available genome sequence.

One of the models for gene conversion at the *pilE* locus implicates the acquisition of *pilS* sequences from the environment [38]. Hence, we investigated whether we could induce recombination at the 3’ end of *tpsA* by exogenously supplying *tpsC* cassettes. In preliminary experiments, we incubated purified chromosomal DNA from strain B16B6 with the homologous (B16B6) or heterologous (α153 and α14) strains. PCR analysis of randomly picked colonies failed to provide evidence for recombination at the 3’ end of *tpsA* (data not shown), indicating that such events are rare consistent with the low recombination frequencies found in vivo. Then, a kanamycin-resistance (*kan*) cassette was inserted between the 5’ end of *tpsC2* and the 3’ end of the TPS island (Figure 4A). When chromosomal DNA of the resulting mutant, designated B16B6Δ*tpsC2-5*, was used to transform the parent strain B16B6, the *kan* cassette was found at the same position as in the original mutant in all 150 transformants examined as evidenced by PCRs. Apparently, the high sequence identity at either side of the *kan* cassette forces reciprocal exchange by homologous recombination. When strain α14 was transformed, recombination had occurred within the 5’ region of the disrupted *tpsC2* cassette of the donor DNA and the *tpsA* gene in the recipient DNA in all 19 kanamycin-resistant transformants examined as revealed in PCRs and subsequent sequence analysis (Figure 4B). Recombination in the various transformants had taken place at a variety of small stretches of sequence identity ranging in size from 5 to 23 bp (Figure S7 in Additional file 1). These results demonstrate that *tpsC* sequences can recombine into the 3’ end of a *tpsA* gene when exogenously supplied and that recombination does not occur at a specific site but can take place at any short stretch of sequence identity within a homologous region.

Next, chromosomal DNA from one of the α14-tpsA* recombinants (#1 in Additional file 1: Figure S7) was used to transform strain α153, which has a different central core region in the *tpsA* gene (in Additional file 1: Figure S2). Two kanamycin-resistant transformants were obtained and PCR analysis and subsequent sequencing (data not shown) revealed that the disrupted *tpsA* of the donor DNA had recombined into the TPS island of α153 (Figure 4C). Thus, not only can *tpsC* cassettes recombine into the 3’ region of *tpsA* genes but also the central region of the *tpsA* gene can be replaced as was observed in vivo in the case of cc11 isolate 348.

## Discussion

TpsA1 proteins of *N. meningitidis* have reported roles in adhesion [16], intracellular survival [17] and biofilm formation [18]. In several other bacteria, TpsA proteins have been described that inhibit the growth of related bacteria probably in competition for the same niche [22,23,25,26]. This CDI activity resides in the C-terminal domain, and an immunity protein encoded by the gene downstream of *tpsA* protects the producing strain. Together, the C-terminal domain of TpsA and the immunity protein function as a toxin/antitoxin pair. We demonstrated here that also the TpsA1 proteins of *N. meningitidis* have growth-inhibitory activity and that the IORF immediately downstream of the *tpsA* gene confers immunity.

Downstream of the *tpsA* gene, the TPS islands of *N. meningitidis* contain a variable number of putative genes, called *tpsC* cassettes, interspersed with IORFs. The *tpsC* cassettes putatively encode N-terminally truncated TpsA homologs, which present entirely different C-terminal domains. It was suggested that these *tpsC* cassettes could recombine with *tpsA* by gene conversion possibly as a mechanism of immune evasion [19-21,31]. Also in several toxin/antitoxin systems, silent genes encoding truncated toxins are present and it was suggested that recombination of these silent genes with the toxin gene alters the CDI mechanism [26]. However, up to date no direct experimental evidence for such recombination events has been reported.

Initial bioinformatic analysis indeed suggested extensive recombination between *tpsA* genes and *tpsC* cassettes, as many cases were found of sequences that are present at the 3’ end of a *tpsC* cassette in one genome and at the 3’ end of a *tpsA* gene in another genome (Figure 1, and Additional file 1: Figure S1 and Table S1). However, because of the different phylogenetic origins of these strains, these observations do not directly prove that recombination does occur in vivo. Therefore, to demonstrate that the expected recombination events indeed occur and to determine their frequency, we turned to the analysis of large collections of strains of the same clonal complexes. Initially, we focused on strains of cc11 and cc8, and we obtained evidence that *tpsC* cassettes can indeed recombine in vivo into the *tpsA* locus, but the observed frequency was dramatically lower than the frequency of antigenic variation by slipped-strand mispairing of other surface structures in the same collection of strains. In cc8 and cc11 isolates, gene conversion at the *pilE* locus is not detectable either [35,36]. This is, however, not due to a general recombination defect also affecting the *tpsA* locus but to the nature of the *pilE* and *pilS* genes present in these strains. *N. meningitidis* strains can elaborate one of two classes of pili. Whereas class I pili are closely related to gonococcal pili, class II pili present remarkably different features, *i.e.* lack of conserved regions in between the semi- and hyper-variable regions, deletion in a hyper-variable region, and no homology to class I pili in the flanking sequences [39]. The cc8 and cc11 strains contain a class II *pilE*[35] and the genome of FAM18 shows only two *pilS* cassettes, which exhibit strong homology to type-I pilin genes [39]. Therefore, *pilE*/*S* recombination is not to be expected and the low recombination frequency at the *tpsA* locus must have a different cause. Consistent with this hypothesis, we also found a very low frequency of recombination in the *tpsA* locus in cc32 isolates, which are proficient in recombination at *pilE*. Considering that the strains we analyzed are from hyperinvasive lineages, the low frequency of recombination at the 3’ end of *tpsA* compared to known mechanisms of antigenic variation suggests that the selection of new TpsA variants in vivo is not driven by immune pressure. Also, although *N. meningitidis* apparently has a large repertoire of alternate cassettes that could replace the killing module at the C terminus of TpsA, it apparently has only a low tendency to change it. This suggests that replacement of the C-terminal cassette of TpsA may even be detrimental to the bacteria. In the nasopharynx, *N. meningitidis* appears in microcolonies [40]. Obviously, within such a microcolony, the substitution of the C terminus of TpsA with the concomitant loss of the immunity protein as found in some cc11 isolates (Figure 3) would be detrimental as the protection against other cells within the community would be lost.

**Figure 5:**
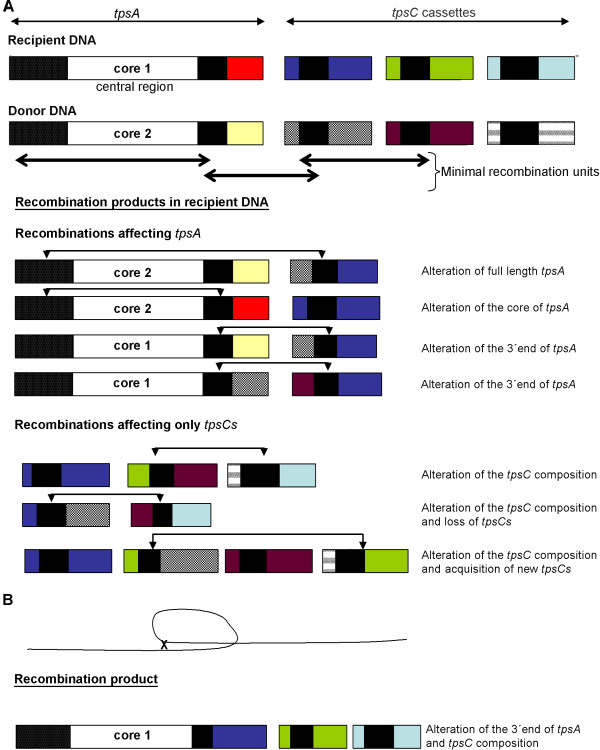
**Recombination model for the TPS system. (A)** Recombination model requiring double crossover. The recipient and donor DNA are indicated in the first and the second line, respectively, and carry in the depicted example a different central core region in *tpsA*. IORFs are not depicted. Within the 3’ region, the *tpsA* genes share sequences with segments at the 5’ end of *tpsC*s (black boxes). The minimal recombination units (indicated by double-headed arrows) are formed by the 5’ region of a *tpsC*, an IORF, and the 5’ region of the immediately downstream *tpsC*. The recombination products show the recipient DNA organization after double crossovers with donor DNA sequences. The double-headed lines show the recombination sites where the crossovers were established. **(B)** Recombination model requiring an intra-chromosomal single crossover and the product of such an event evolved from the organization of the recipient DNA shown in the first line of panel A.

Although the frequency is low, the postulated recombination at the 3’ end of *tpsA* was confirmed both in vivo and in vitro. Figure 5A illustrates a model for the recombination mechanism as deduced from our work. We postulate that the minimal recombination unit consists of a *tpsC*, an IORF and the 5’ region of the next *tpsC*. Homologous recombination occurs in the borders of the recombination unit in the donor DNA with equivalent sequences in the recipient DNA. During the recombination event, a variable number of additional *tpsC*s, together with their downstream IORFs, can be incorporated or lost in the recipient DNA, which may result in extensive modification of the *tpsC* repertoire and may or may not alter the 3’ end of the *tpsA* gene. The sequences of the IORFs located immediately downstream of *tpsA*s or *tpsC*s with similar 3’ ends are highly conserved and show no sequence similarity with other IORFs. In the recombination models, the IORFs always remain associated with the upstream *tpsC* and will therefore confer immunity to the producing strain when this *tpsC* is recombined into the *tpsA* expression locus (Figure 3). Besides this process, the central region of the *tpsA* can also be replaced in which case the recombination at the 5’ end occurs within the DNA encoding the TPS domain or in *tpsB* or even further upstream, and at the 3’ end in the 5’ region of a downstream *tpsC*. No functional activity has been assigned to this central core region. Perhaps, it is only required for the efficient secretion and surface exposure of the killing module. However, considering its size and variability, it may serve additional functions. Besides mediating growth inhibition, meningococcal TpsA proteins are also involved in adhesion [16], intracellular survival [17] and biofilm formation [18]. Further experiments should clarify if the core region has a direct role in these additional TpsA activities.

*Neisseria* shows autolytic behavior and has the capacity to take up *Neisserial* DNA fragments by recognition of specific DNA uptake sequences [38]. The acquisition of *pilS* sequences from the environment is used for recombination of *pilS* to *pilE*[38,41]. The presence of DNA uptake sequences within the TPS islands, mostly located at the 5’ end of *tpsC* cassettes (Additional file 1: Figure S1), is consistent with the hypothesis that donor DNA for recombination in *tpsA* can be acquired from the medium. Our in vitro recombination studies offer experimental support for this mechanism. Obviously, for exchange of the central core region of *tpsA*, uptake of donor DNA from the environment is a necessity. Another model explains *pilE/S* gene conversion based on intracellular recombination substrates and assumes that DNA replication producing two chromosomes precedes recombination by double crossover [41,42]. Yet another model implicates the generation of an extrachromosomal circular intermediate generated by a single crossover (Figure 5B) [42,43]. Both models could explain the TPS organization in cc11 isolates in which the *tpsC1* cassette has apparently recombined into the *tpsA* locus (Figure 3A). It is interesting to note that *tpsC5* of FAM18 is the most distal *tpsC* in many TPS regions (colored blue in Figure S1in Additional file 1). The absence of a downstream *tpsC* cassette for establishing recombination may prevent its movement to another position in the *tpsC* repertoire. In the genome of strain α14, this *tpsC* has recombined into the *tpsA* gene with concomitant loss of all intervening *tpsC*s (Figure S1 in Additional file 1), which can be explained through a single crossover recombination event*.* Thus, like in the *pilE*/*pilS* system [13], recombination of *tpsC*s into the *tpsA* locus can be mediated by three different mechanisms involving uptake of DNA from the environment, recombination between sister chromosomes, or an intra-chromosomal single crossover event.

Where exactly the recombination between *tpsA* and *tpsC* cassettes takes place, has remained unclear thus far. Based on in silico analysis, it was previously predicted to be mediated by multiple direct repeats located in the 3’ half of *tpsA* and the 5’ end of *tpsCs*[21]. In contrast, Poole and colleagues suggested that these events take place at a more specific location, i.e. the VENN domain that is present in many *tpsA*s and *tpsC*s [26], including *tpsA* and *tpsCs* of *N. meningitidis* (indicated in Additional file 1: Figure S3 ). The results presented here show that the recombination can take place in small shared stretches of different length (5 to 23 bp, in Additional file 1: Figure S7) that are broadly distributed along a larger homology domain (in Additional file 1: Figure S3), in line with what was reported in the *pilE/S* system [13].

Although the postulated recombination at the 3’ end of TpsA was demonstrated in this study, the frequency was very low. If the recombination frequency is that low and recombination may even be detrimental, why have most *N. meningitidis* isolates generated such a large collection of *tpsC* cassettes? Possibly, the importance of the collection is not in the *tpsC* cassettes, but in the associated IORFs that, together, confer immunity to a large variety of TpsAs produced by competitors in the nasopharynx. Consistent with the proposed minor role of the *tpsC* cassettes is that we detected many *tpsC*s with internal stop codons, frameshift mutations or extensive truncations at the 3’ end removing the homology domain for recombination into the *tpsA* locus (Additional file 1: Table S1 and Figure S1).

## Conclusions

Several TpsA proteins produced by Gram-negative bacteria inhibit the growth of other related bacteria probably in competition for the same niche. The toxic activity resides in its C-terminal region while an immediately downstream located IORF encodes an antitoxin protein. Here, we showed that also meningococcal TpsA mediates growth inhibition and that the downstream IORF confers immunity to the producing strain. We also demonstrated that *tpsC* cassettes can recombine into the *tpsA* locus leading to the presentation of a different toxic module at the C terminus of TpsA. We obtained evidence for such recombination both in vivo during infection and under laboratory conditions. However, new TpsA variants are generated only at a very low frequency possibly due to negative selection against recombination as consequence of the concomitant loss of the immunity protein resulting in sensitivity to the TpsA protein produced by the congeners in the same microbial community. The *tpsC* cassettes for recombination can be acquired from the environment and recombine into the *tpsA* gene or in the downstream region, potentially resulting in an increased or decreased number of *tpsC* cassettes and associated IORFs. We propose that the main goal of recombination is the collection of new IORFs for protection against a variety of TpsA proteins. Further studies on the mechanisms of TpsA-mediated fratricide might eventually enable us to fight these pathogens with their own weapons.

## Methods

### Bacterial strains and growth conditions

A panel of 424 *N. meningitidis* strains was examined here (Additional file 2). Disease isolates were from The Netherlands Reference Laboratory for Bacterial Meningitis (NRLBM) in Amsterdam and carrier strains from the Institut für Hygiene und Mikrobiologie, Würzburg. Isolates were classified in clonal groups based upon sequence analysis of conserved house-keeping genes and comparison of the results with the data on the Neisseria Multi Locus Sequence Typing website (http://pubmlst.org/neisseria/) [44]. Included in this study were also reference strains B16B6 [45], 2996 [46], FAM18 [2], α14 and α153 [27] (Additional file 2). Strains were grown at 37°C on GC medium (Oxoid) supplemented with Vitox (Oxoid) and antibiotics as appropriate (erythromycin 7 μg/ml; kanamycin, 100 μg/ml; chloramphenicol, 5 μg/ml; rifampicin, 50 μg/ml) in candle jars or in tryptic soy broth (TSB; Beckton Dickinson) at 37°C with constant shaking at 110 rpm for 7 to 8 hours. Bacteria were inactivated for 30 minutes at 56°C when appropriate. *E. coli* strain DH5α was grown in LB medium containing 100 μg/ml of kanamycin or ampicillin when required.

### PCR amplification and sequencing

Bacteria, grown for 12 hours on plate, were resuspended in de-ionized water and boiled for 10 minutes. The supernatant obtained after removal of the cell debris by centrifugation was used as template DNA for PCRs [19]. PCRs were performed using 2 μl of extracted DNA, 0.25 μM of different primer combinations (Additional file 3), 0.5 U *Taq* DNA polymerase, 200 μM dNTPs and PCR buffer (all from Promega). For PCR products to be sequenced, the Expand High Fidelity PCR System (Roche) was used. PCR conditions consisted of 10 minutes incubation at 95°C, 30 cycles of 1 minute at 95°C, 0.5 minutes at 58°C, and 2.5 minutes at 72°C, and finally 10 minutes incubation at 72°C. The PCR products were analyzed on 1% agarose gels and visualized with ethidium bromide.

For sequencing of the TPS islands, several overlapping DNA fragments of variable length (568–2500 bp) were amplified, purified using the Wizard SV Gel and PCR Clean-Up System (Promega), and sequenced using internal primers at Macrogen (Seoul, Korea). When required, PCR products were cloned into the TOPO TA vector (Invitrogen). The plasmids were purified with the plasmid extraction kit I (Omega) and the inserts were sequenced using M13 universal primers. PCR reactions were carried out independently and repeated at least twice for each fragment. Sequences were assembled using the SeqMan II software (DNAstart Inc.).

For assessment of gene conversion at the *pilE* locus, we first determined the presence of the *pilE* locus by PCR with primers 5’pilE and 3’pilE, which anneal upstream and downstream of *pilE*, respectively [35]. In a second PCR, we combined primer 5’pilE with a primer (TTAGCTGGCATCACTTGCG) that anneals with a hypervariable region of *pilE* in MC58.

### DNA sequence analyses

The *tpsA* and *tpsC* sequences available in the genome sequence databases of *N. meningitidis* strains α14, α153, α275 [27], 053442 [28], 8013 [29], FAM18 [2], MC58 [30], and Z2491 [31] were used. Comparative analysis within TPS regions was performed using Clone Manager software. All sequences were aligned using MAFFT server version 6 (http://align.bmr.kyushu-u.ac.jp/mafft/online/server/). Amino-acid distances were determined by phylogenetic analysis using the Neighbor-joining method with the available MEGA software version 4.0 (http://www.megasoftware.net/).

### Generation of DNA constructs and mutants

To obtain a Δ*tpsA-tpsC* knockout mutant, DNA fragments *h1*, corresponding to the *tpsB* gene, and *i*, located downstream of the TPS island, were amplified from chromosomal DNA of strain B16B6 using the primers listed in Additional file 3. Both PCR products were purified, digested with proper restriction enzymes (Additional file 3), and sequentially cloned into pUC21. Then, a *kan* cassette was inserted between the cloned fragments yielding plasmid pKOtpsA-tpsC. To obtain the Δ*tpsC2-5* mutant, DNA fragment *h2*, encompassing most of *tpsC2* and its upstream IORF, was amplified, purified, and exchanged for fragment *h1* on pKOtpsA-tpsC using restriction enzymes NotI and DraIII yielding plasmid pKOtpsC2-5. Plasmid pKO-tpsB1 [19] was used to obtain a *tpsB* mutant. The knockout constructs were purified with the plasmid extraction kit I and used to transform [47] strain B16B6 where the target genes were disrupted by homologous recombination. Appropriate PCRs were used to verify the presence of the correct mutations in kanamycin-resistant transformants and, where appropriate, the loss of TpsA or TpsB expression was confirmed on Western blots.

The unencapsulated mutant of serogroup B strain B16B6, designated BB-1, was previously described [48]. Unencapsulated mutants of derivatives of B16B6 and of serogroup C isolate 2001044 were obtained by gene replacement as described [48]. Recombination of the knockout constructs into the chromosome resulted in replacement of the complete capsule locus or only the *siaD* gene by an erythromycin- or chloramphenicol-resistance cassette, respectively. Correct mutations were verified by appropriate PCRs. The absence of serogroup B capsule in the B16B6 derivatives was also confirmed with monoclonal antibody anti-MBPS [49].

For complementation experiments, DNA fragment *v* corresponding to the IORF downstream of *tpsA* was amplified from chromosomal DNA of strain B16B6 using the primers listed in Additional file 3: Table S3. The PCR product was purified and cloned via NdeI and AatII digestion into pEN11-Imp [50] to yield plasmid pFPIORF_1_. The correct sequence of the inserted DNA fragment was confirmed.

### Electrophoresis and immunoblotting

For SDS-PAGE, 8% (w/v) polyacrylamide gels were used. Proteins were visualized with Coomassie Brilliant Blue G250 or transferred to nitrocellulose membranes. The blots were incubated with blocking buffer [phosphate-buffered saline with 0.5% (w/v) non-fat dried milk (Protifar, Nutricia) and 0.1% (v/v) Tween 20 (Merck)]. Washes were done with blocking buffer without Protifar. All incubations were carried out for 1 hour at room temperature with constant shaking. After incubation with polyclonal antiserum directed against the TPS domain of TpsA1 [19] or NalP (anti-1669) [51] or with monoclonal antibodies for the specific detection of OpaJ and OpaA (15–1.P5.5) [52] or OpaD and OpaB (MN20E12.70) [53] at working dilutions in blocking buffer, the blots were developed with horseradish-peroxidase-conjugated goat anti-rabbit-IgG or anti-mouse-IgG (Biosource International) and the Pierce ECL Western Blotting Substrate.

### Growth inhibition assay

The unencapsulated derivative of strain B16B6, BB-1, was transformed with plasmid pEN300 [51] containing a chloramphenicol-resistance marker to be able to discriminate between bacteria from co-cultures. A spontaneous rifampicin-resistant derivative of BB-1 was used for the same purpose in complementation assays, where the target cells carried a chloramphenicol-resistance marker on plasmid pFPIORF_1_. Bacteria from overnight cultures were inoculated in antibiotic-free TSB either containing or not 0.25 mM IPTG and grown with shaking until they reached an optical density at 600 nm of ~3. Subsequently, bacteria were mixed 1:1 and drops were spotted on antibiotic-free GC medium plates either with or without 0.25 mM IPTG and incubated at 37°C in candle jars in a humid atmosphere containing ~5% CO_2_. After different time periods, the bacteria were scraped from the plates, resuspended in TSB, and the ratio of the different bacteria in the co-culture was determined by plating on selective GC plates containing the appropriate antibiotics and overnight incubation. The numbers of viable cells in the initial cultures were similarly determined.

## Abbreviations

Bp: Base pairs; CDI: Contact-dependent growth inhibition; IORF: Intervening open reading frame; IPTG: Isopropyl-β-D-1-thiogalactopyranoside; Kan: Kanamycin-resistance; ORF: Open reading frame; PCR: Polymerase chain reaction; TPS: Two-Partner Secretion; TSB: Tryptic soy broth.

## Competing interests

The authors declare they have no competing interests.

## Authors’ contributions

JA, PvU, AvdE, and JT conceived and designed the experiments; JA and KS performed the experiments; JA, AvdE, and JT analyzed the data; JA and JT wrote the paper. All authors have read and approved the manuscript.

## Supplementary Material

Additional file 1 Supplemental information containing one supplemental table and seven supplemental figures. Click here for file

Additional file 2: Table S2 listing the meningococcal isolates used in this study and summarizing the results of PCR and Western blot analyses. Click here for file

Additional file 3: Table S3 listing the PCR primers used in this study. Click here for file
