# Supplementary material for: Domain exchange at the 3’ end of the gene encoding the fratricide meningococcal two-partner secretion protein A
Source: BMC Genomics. 2013 Sep 14;14:622. doi: 10.1186/1471-2164-14-622 (PMC3848433; doi:10.1186/1471-2164-14-622)
Supplement: Additional file 1 — Supplemental information containing one supplemental table and seven supplemental figures. [file 1471-2164-14-622-S1.pdf]

Table S1 Sequence similarities in the C-terminal regions of TpsA and TpsC polypeptides of *N. meningitidis*[a]

Locus tag[b]	Genome	Identity (aa)[c]	Genes[d]
**NMC0444**	**FAM18**	**100%**	***tpsA***
NMCC_0499*	053442	100%	*tpsA*
NMB0497	MC58	100%	*tpsA*
NMV_1919	8013	100%	*tpsA*
**NMC0446**	**FAM18**	**100%**	***tpsC1***
NMB1779	MC58	100%	*tpsA*
NME_0860	α153	100%	*tpsA*
NMV_1917	8013	100%	*tpsC1*
**NMC0448**	**FAM18**	**100%**	***tpsC2***
NMB0509	MC58	100%	*tpsC4*
NMV_0571	8013	100%	*tpsC5*
NMW_2275	α275	100%	*tpsC1*
**NMC0450**	**FAM18**	**100%**	***tpsC3***
NMA0688	Z2491	100%	*tpsA*
NMV_0558	8013	100%	*tpsC1*
**NMC0452**	**FAM18**	**100%**	***tpsC4***
NMA0692	Z2491	92% (138)	*tpsC2*
NMB0504/0506	MC58	93% (139)	*tpsC3*
NMV_0560/0562*	8013	95% (143)	*tpsC2*
NMV_0580#	8013	100% (56)	*tpsC9*
**NMC0456**	**FAM18**	**100%**	***tpsC5***
NMO_0398	α14	94% (141)	*tpsA*
NMA0695	Z2491	93% (139)	*tpsC3*
NMB0514/0515*	MC58	99% (149)	*tpsC6*
NMCC_0457*	053442	94% (141)	*tpsC3*
NMV_0575#	8013	90% (22)	*tpsC7*
NMV_0577#	8013	90% (22)	*tpsC8*
NMV_1913#	8013	100% (32)	*tpsC3*
NMW_2272#	α275	100%	*tpsC3*
NMW_2270.1#	α275	90%(22)	*tpsC4*
**NMA0690**	**Z2491**	**100%**	***tpsC1***
NMB0502	MC58	99% (149)	*tpsC2*
NMV_0565	8013	98% (147)	*tpsC3*
**NMB1772**	**MC58**	**100%**	***tpsC2***
NMV_0573*	8013	100%	*tpsC6*
NMV_1915*	8013	100%	*tpsC2*
**NMB0499**	**MC58**	**100%**	***tpsC1***
NMB1775	MC58	100%	*tpsC1*
NMCC_0451	053442	99% (149)	*tpsC1*
NMV_0567	8013	100%	*tpsC4*
**NMB0511**	**MC58**	**100%**	***tpsC5***
NMCC_0454	053442	100%	*tpsC2*
NMW_2274	α275	100%	*tpsC2*
**NMV_0556**	**8013**	**100%**	***tpsA***
NMW_2276	α275	99%	*tpsA*

[a] Representative TpsA and TpsC amino-acid sequences are grouped based on sequence similarity ≥ 90% in the C-terminal region. Identity between sequences of different

groups is < 20%. The Table is not exhaustive and includes only *tpsA* genes and *tpsC* cassettes that are used in Figure S1. The sequence of the C-terminal domain of the first protein of each group (bold) was used as query in initial searches.
[b] The *tpsC* cassettes and a *tpsA* gene disrupted by stop codons, frame shifts or a transposase gene are indicated with asterisks. Such interruptions were removed for optimal alignment. Small, often non-annotated *tpsC* cassettes that lack a homology domain for recombination into the *tpsA* locus are indicated with a # sign. Double numbering means either that two annotated ORFs together constitute a *tpsC* cassette that is disrupted by a frame shift mutation or that additional ORFs are annotated within the same ORF as the *tpsC* cassette.
[c] Sequence identity is based on a C-terminal stretch of 150 amino-acid residues or a lower number if indicated in parentheses.
[d] The *tpsC* cassettes are sequentially numbered according to their order downstream of the *tpsA* gene in the corresponding TPS island.

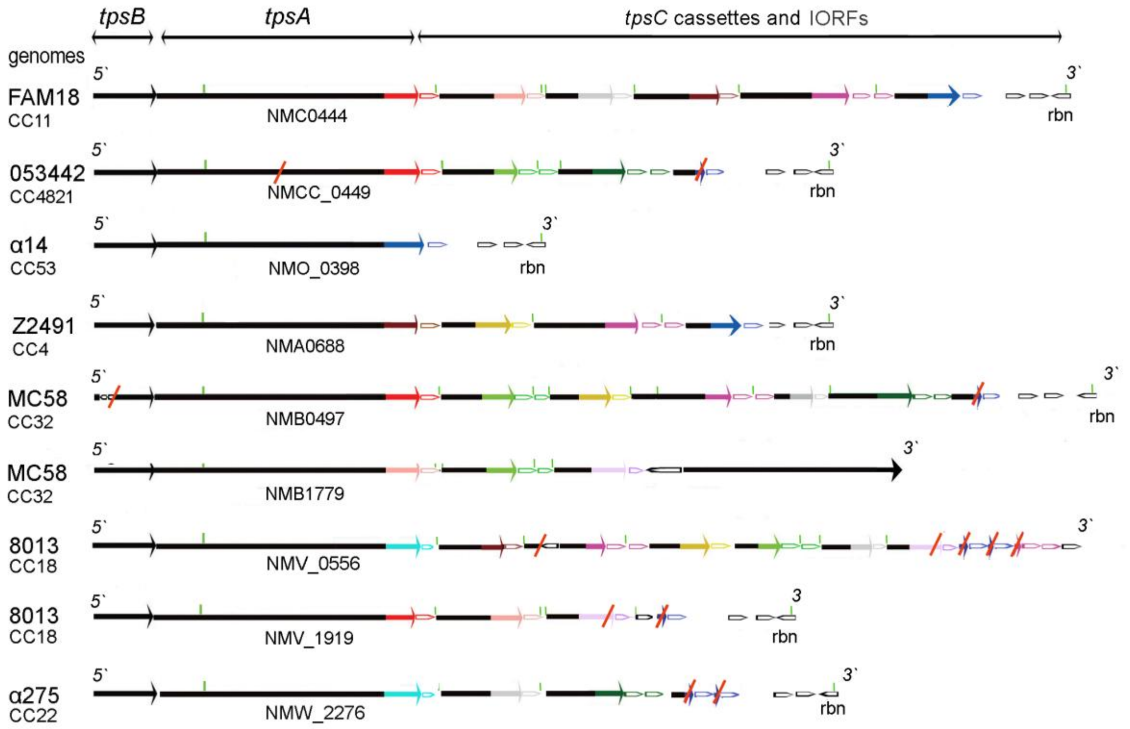

Figure S1 Organization of TPS islands in available *N. meningitidis* genome sequences. The TPS islands containing *tpsA1* genes (indicated by their locus tags) of seven strains of different clonal complexes are shown. The organization of the TPS island in strain α153 could not be depicted since it is not located on a single contig in the incomplete genome sequence. The 3' ends of *tpsA* genes and *tpsC* cassettes with high sequence similarity are colored identical in the different islands. The IORFs located in between the *tpsA* genes and *tpsC* cassettes are indicated with open arrows. Their sequences are highly divergent, but *tpsA*s and *tpsC*s with high sequence similarity in their C-terminal end are always followed by IORFs with high sequence similarity. The *tpsA* of strain 053442 contains a stop codon at codon position 1350 (indicated with a red slash). Also several *tpsC* cassettes are disrupted by stop codons, frame-shift mutations or extensive deletions at the 3' end (red slashes). The positions of DNA-uptake sequences are given as green dashes. Note that several *tpsC* cassettes and IORFs depicted here are not annotated in the genome sequences but were identified by our own analysis of the sequences.

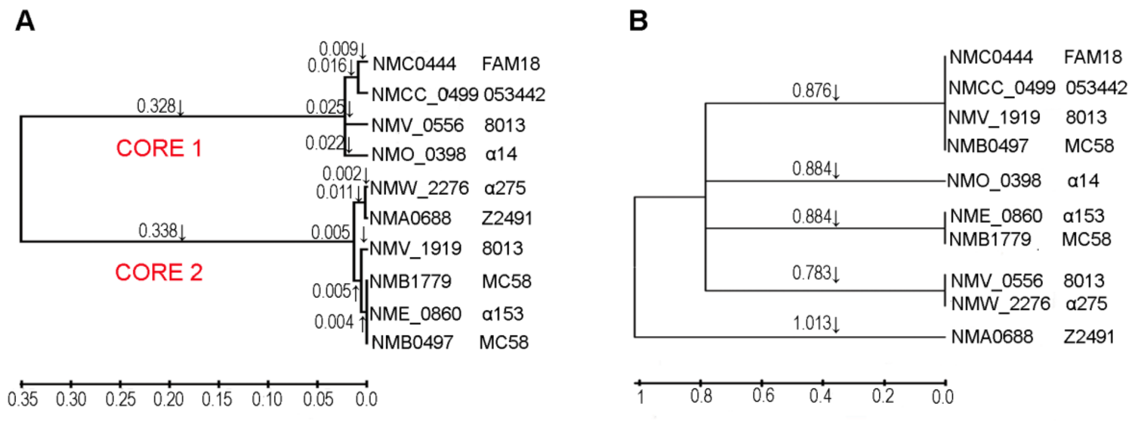

Figure S2 Characterization of *tpsA1* genes and *tpsC* cassettes. Phylogenetic trees based on the amino-acid sequences of (A) the central core region and (B) the last 150 residues of the TpsA proteins. These trees show heterogeneous divergence distances in the central and C-terminal regions. A premature stop codon in *tpsA* of strain 053442 was eliminated for inclusion of this TpsA in the analysis.

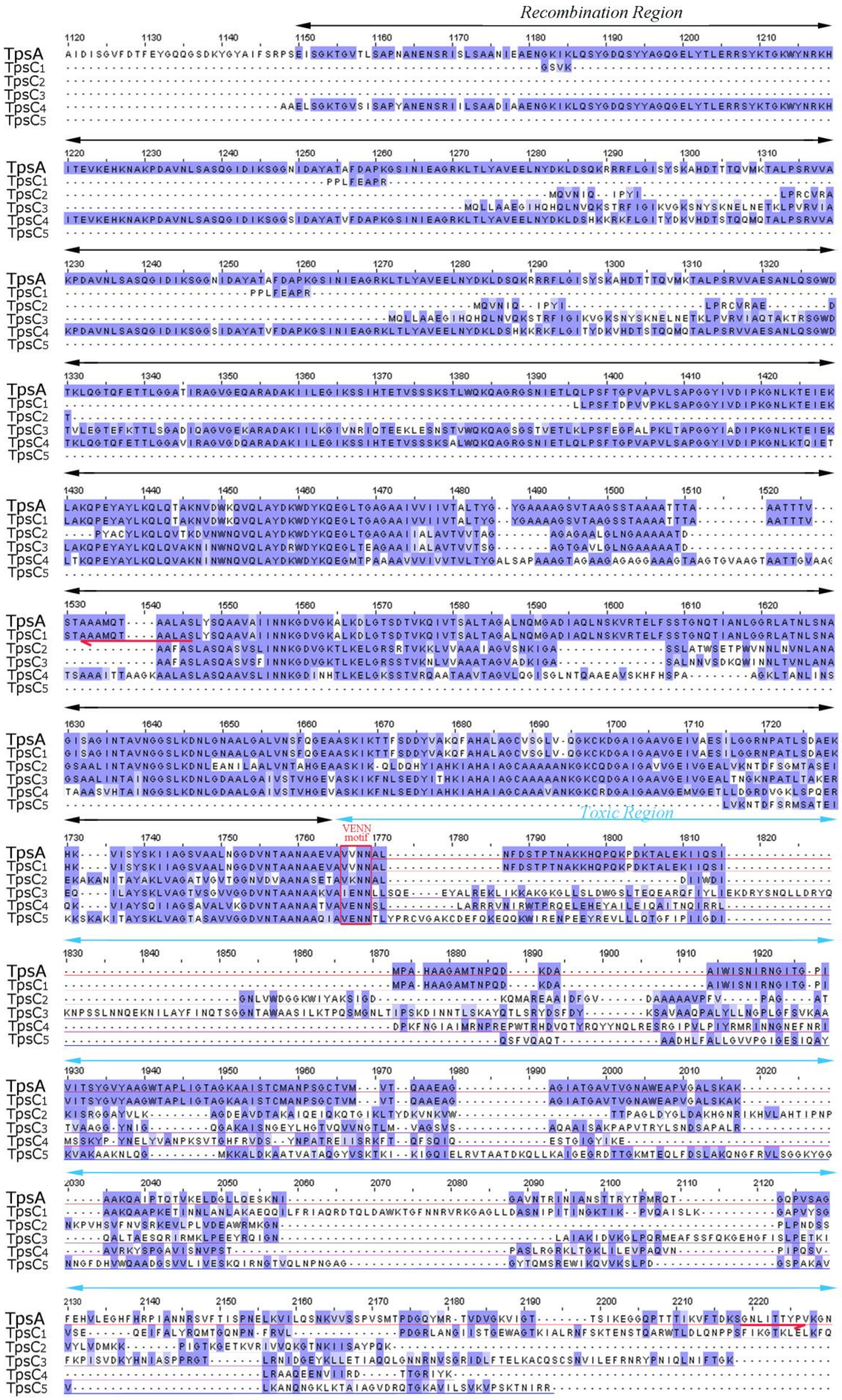
Recombination Region
Toxic Region
VENN motif
TpsA
TpsC1
TpsC2
TpsC3
TpsC4
TpsC5

Figure S3 Alignment of predicted protein sequences of the C-terminal region of TpsA and full-length TpsCs of FAM18. The alignment was generated with Mafft software and visualized by Jalview 2.8. Identical residues in the alignment are indicated with similar color shading. A previously identified conserved VENN motif (22) is indicated in a red box. The toxic module in *E. coli* TpsA proteins (indicated by a light blue double-headed arrow above the sequences) was shown to be located downstream of this conserved motif (22). This domain is highly divergent among the sequences displayed (which are underlined in the same color coding as in Figure 1) except that TpsC1 still displays considerable homology to TpsA in the N-terminal part of the domain. Upstream of the VENN domain are large stretches of homology between the sequences, which could be used for recombination between *tpsC*s and *tpsA* to replace the toxic module in TpsA (indicated by a black double-headed arrow). The relative positions of the primers used to generate PCR fragment *a1* in the screens for recombination at the 3’ end of *tpsA* (Figure S4) are also indicated (red arrows) to demonstrate that they are located in regions of low homology.

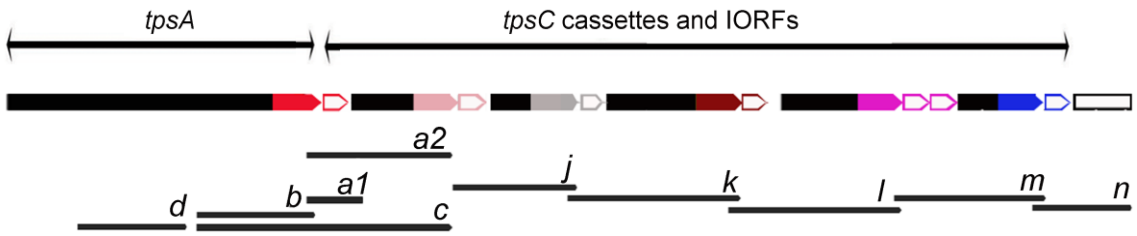

Figure S4 Analysis of the organization of the TPS island in isolates of cc11 and cc8 by PCR. The genetic organization of the TPS island of cc11 strain FAM18 is depicted as in Figure 1. DNA fragments targeted by PCR to determine the organization of the TPS islands in various isolates are indicated underneath the genetic organization and the corresponding primers are listed in Table S3 in Additional file 3. The results for individual isolates are summarized in Table S2 in Additional file 2. In total, 274 out of 277 cc11 disease isolates, 6 out of 6 cc11 carrier isolates and 91 out of 91 disease isolates of cc8 were positive for fragment *a1*. Various isolates that were positive for *a1* were analyzed further. In total, 48 disease isolates of cc11 were tested for fragments *b*, *c* and *d*, and found to be positive. The six carrier isolates of cc11 were tested for *a2* and *b* and found positive. Eight disease isolates of cc8 were tested for *b* and *d* and found positive. Strain 2996, also of cc8, was tested for *a1*, *a2*, *b*, and *j*-*m* and found positive. The three isolates of cc11 that were negative for *a1* (i.e. 2001044, 2020041 and 348) were also further analyzed. Isolates 2001044 and 2020041 did yield an amplicon for fragment *c*, which was, however, ~ 3 kb smaller than the corresponding one in FAM18 suggesting a large deletion in the corresponding DNA fragment that could result from the recombination of the *tpsC1* cassette into the *tpsA* locus. Isolate 348 was negative for fragment *c* but positive for fragment *g*, which is obtained with primers annealing in the *tpsA* and *tpsC1* genes of strain Z2491 (not depicted in the figure). All isolates were also positive for fragments *j*-*n*.

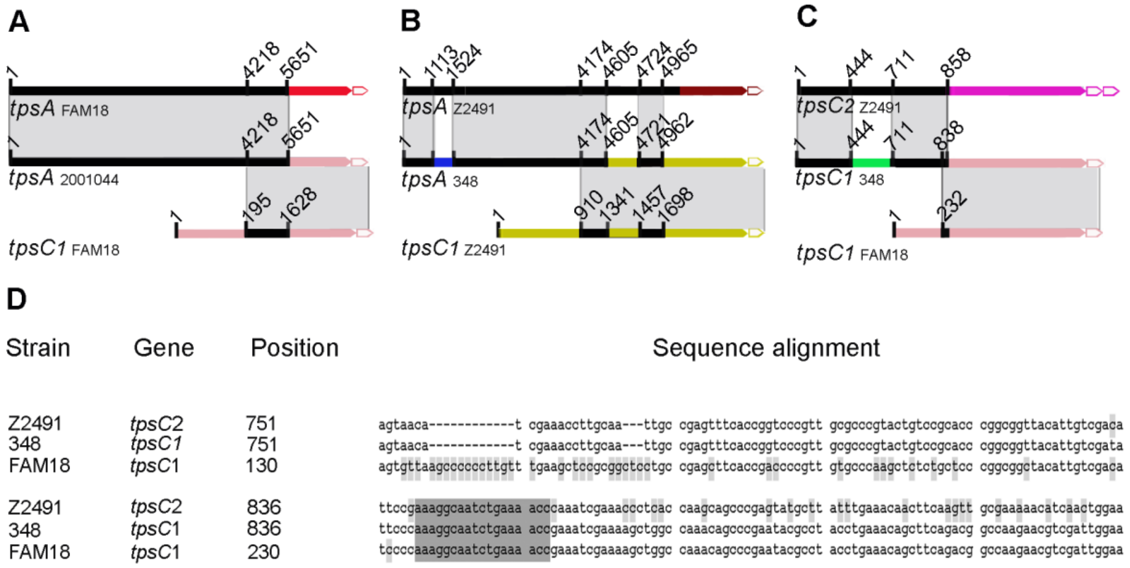

Figure S5 Identification of recombination sites in *tps* genes of deviant cc11 isolates. (A) The *tpsA* of isolate 2001044 (middle) was generated by recombination of genes similar to the *tpsA* (top) and *tpsC1* (bottom) from strain FAM18. Regions of high sequence identity are indicated by grey shading. A region corresponding to bp 4218-5651 in *tpsA* of isolate 2001044 is shared in the three genes. Comparison of the upstream and downstream sequences suggests that recombination took place in this region. (B) The *tpsA* of isolate 348 (middle) was generated by recombination of genes similar to the *tpsA* (top) and *tpsC1* (bottom) from strain Z2491. Recombination apparently took place in the region that corresponds to bp 4174-4605 in *tpsA* of isolate 348. (C) The *tpsC1* of isolate 348 (middle) was generated by recombination of genes similar to *tpsC2* of strain Z2491 (top) and *tpsC1* from strain FAM18 (bottom). Recombination apparently took place in a short stretch of sequence identity corresponding to bp 838-858 in *tpsC1* of isolate 348, which is shown in detail in panel D. (D) Alignment of relevant parts of the nucleotide sequences of *tpsC2* of strain Z2491, *tpsC1* of FAM18 and the recombinant *tpsC1* of isolate 348. Upstream and downstream of a shared 18-bp stretch of sequence identity (dark grey), the *tpsC1* of isolate 348 is similar to *tpsC2* of Z2491 and *tpsC1* of FAM18, respectively. Deviations from the *tpsC1* sequence of isolate 348 in the upstream and downstream regions, respectively, are highlighted by light grey shading.

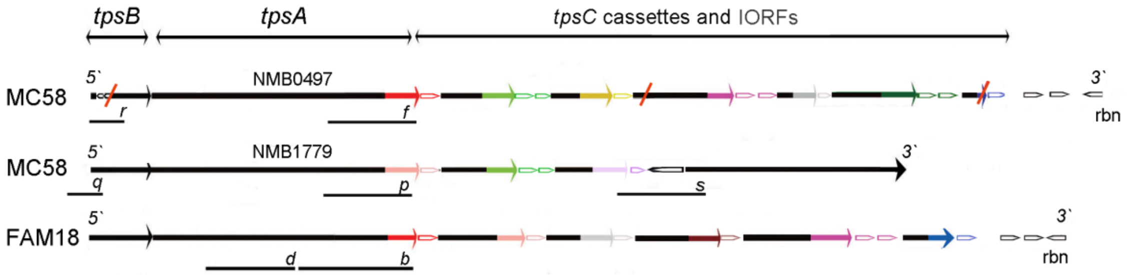

Figure S6 Analysis of the organization of the TPS islands in isolates of cc32. The genetic organization of the two TPS islands containing *tpsA1* genes (*tpsA1b* with locus tag NMB0497 and *tpsA1a* with locus tag NMB1779) of cc32 strain MC58 and that of cc11 strain FAM18 is depicted. DNA fragments targeted by PCR to determine the organization of the TPS islands in the cc32 isolates are indicated underneath the genetic organization. All isolates yielded an amplicon for fragment *f*, demonstrating conservation of the *tpsA1* locus corresponding to NMB0497. However, 13/50 isolates were negative for fragment *p* suggesting that either recombination had occurred at the *tpsA1* locus corresponding to NMB1779 or the *tpsA1* locus was not duplicated in these isolates. The latter possibility was tested in PCRs targeting fragments *q*, *r* and *s*, which cover the boundaries of the TPS islands [19]. In 11 isolates, only fragment *q* was amplified indicating that the genome rearrangement that leads to duplication of *tpsA1* had not occurred [19]. The other two isolates (isolates 2081107 and 2061468) were positive in all three PCRs. Isolate 2081107 was also positive in PCRs for fragments *b* and *d*, suggesting the presence of a *tpsA1* similar to that of FAM18, which has a central core different from that of the *tpsA1* genes of MC58. Therefore, we hypothesize that this *tpsA* was acquired by horizontal gene transfer in this isolate. To identify the recombination that had occurred in the second *tpsA1* of isolate 2061468, we performed a series of PCRs with the forward primer annealing with the core of NMB1779 (*i.e.* the forward primer for fragment *p*) and reverse primers annealing in the 3' regions of the *tpsC* cassettes in MC58. We obtained a PCR product (labeled *t* in Table S3 in Additional file 3) of ~2300 bp with the reverse primer annealing to the *tpsC5* downstream of NMB0497, indicating that this *tpsC* had recombined into the *tpsA1* corresponding to NMB1779.

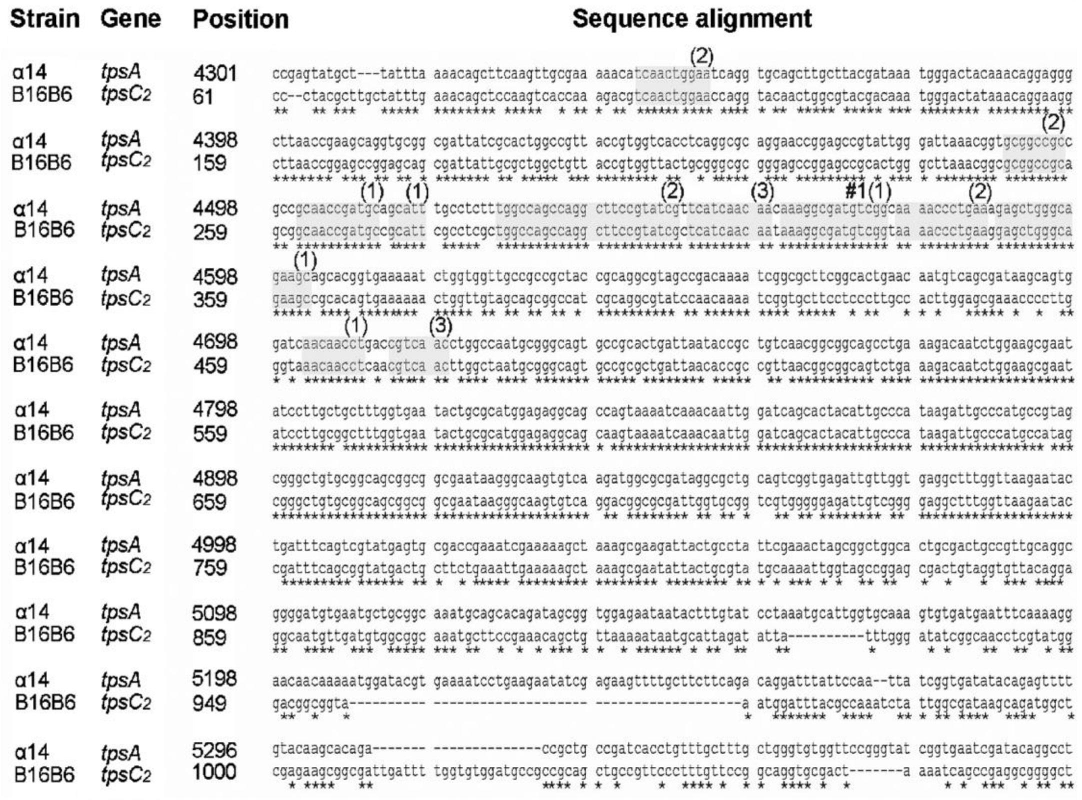

Figure S7 Recombination sites in α14-tpsA* recombinants obtain after transformation of strain α14 with chromosomal DNA of strain B16B6Δ*tpsC2-5*. An alignment of sequences of *tpsA* of α14 and *tpsC2* of B16B6 is shown. Positions of nucleotides relative to the start codon of the ORFs are indicated. Identical residues in the alignment are indicated with asterisks. Relevant DNA segments of the *tpsA* genes of 19 α14-tpsA* recombinants were sequenced and the recombination sites, highlighted in grey boxes, were identified by sequence identity in the upstream region with *tpsA* of α14 and in the downstream region with *tpsC2* of B16B6. The numbers of transformants with the same recombination site are indicated within parentheses. #1 indicates the recombination site in the clone that was used for further experiments.
