# Supplementary material for: Domain exchange at the 3’ end of the gene encoding the fratricide meningococcal two-partner secretion protein A
Source: BMC Genomics. 2013 Sep 14;14:622. doi: 10.1186/1471-2164-14-622 (PMC3848433; doi:10.1186/1471-2164-14-622)
Supplement: Additional file 2: Table S2 — listing the meningococcal isolates used in this study and summarizing the results of PCR and Western blot analyses. [file 1471-2164-14-622-S2.pdf]

Table S2. Meningococcal isolates used in this study and results of PCR and antisera reactions

Isolate[a]	Isolation year	Geographic origin	Source[b]	Serogroup	ST	Clonal Complex	PCR[c]	Anti sera[d]
B16B6[#]	1960	United States	Invasive	B	11	11	a1,b,c,d	nd
FAM18[#]	1983	United States	CSF	C	11	11	a1,b,c,d	nd
59	1960	The Netherlands	CSF	B	11	11	a1	nd
69	1960	The Netherlands	CSF	B	11	11	a1,b,c,d	a, b
102	1960	The Netherlands	CSF	B	11	11	a1	nd
115	1960	The Netherlands	CSF	B	11	11	a1	nd
142	1961	The Netherlands	CSF	C	11	11	a1	nd
156	1961	The Netherlands	CSF	B	6010	11	a1	nd
175	1961	The Netherlands	CSF	B	6015	11	a1	nd
177	1961	The Netherlands	CSF	B	11	11	a1	nd
187	1961	The Netherlands	CSF	B	11	11	a1	nd
192	1962	The Netherlands	CSF	C	6824	11	a1	nd
197	1962	The Netherlands	CSF	C	11	11	a1,b,c,d	a
222	1962	The Netherlands	CSF	B	6881	11	a1	nd
295	1963	The Netherlands	CSF	C	6831	11	a1	nd
307	1963	The Netherlands	CSF	B	11	11	a1	nd
314	1963	The Netherlands	CSF	C	6833	11	a1	nd
326	1963	The Netherlands	Blood	C	11	11	a1,b,c,d	
328	1963	The Netherlands	CSF	B	11	11	a1	nd
348	1963	The Netherlands	CSF	C	11	11	g	b
354	1963	The Netherlands	CSF	C	11	11	a1	nd
382	1963	The Netherlands	CSF	C	11	11	a1,b,c,d	b
385	1963	The Netherlands	CSF	C	11	11	a1	nd
399	1963	The Netherlands	CSF	B	11	11	a1	nd
413	1963	The Netherlands	CSF	C	11	11	a1	nd
422	1964	The Netherlands	Blood	C	11	11	a1,b,c,d	
423	1964	The Netherlands	CSF	C	4706	11	a1	nd
427	1964	The Netherlands	CSF	B	11	11	a1	nd
433	1964	The Netherlands	CSF	C	11	11	a1	nd
435	1964	The Netherlands	CSF	B	11	11	a1	nd
442	1964	The Netherlands	CSF	C	11	11	a1	nd
448	1964	The Netherlands	CSF	C	11	11	a1,b,c,d	
450	1964	The Netherlands	Blood	C	11	11	a1	nd
455	1964	The Netherlands	Blood	C	4706	11	a1	nd
461	1964	The Netherlands	CSF	B	11	11	a1,b,c,d	
470	1964	The Netherlands	CSF	C	11	11	a1	nd
475	1964	The Netherlands	CSF	C	11	11	a1	nd
479	1964	The Netherlands	Blood	C	11	11	a1	nd
851100	1985	The Netherlands	CSF	B	11	11	a1	nd
851452	1985	The Netherlands	CSF	B	11	11	a1,b,c,d	a, b
900106	1990	The Netherlands	CSF	B	11	11	a1	nd
900234	1990	The Netherlands	CSF	B	11	11	a1	nd
900707	1990	The Netherlands	CSF	C	11	11	a1,b,c,d	a, b
901140	1990	The Netherlands	CSF	C	11	11	a1	nd
902208	1990	The Netherlands	CSF	C	11	11	a1	nd
902286	1990	The Netherlands	CSF	C	2510	11	a1	nd
990056	1999	The Netherlands	CSF	C	51	11	a1,b,c,d	
990602	1999	The Netherlands	CSF	C	11	11	a1	nd

990738	1999	The Netherlands	CSF	C	11	11	a1	nd
990797	1999	The Netherlands	CSF	C	5450	11	a1	nd
990947	1999	The Netherlands	CSF	C	11	11	a1,b,c,d	a
991044	1999	The Netherlands	CSF	C	11	11	a1	nd
991174	1999	The Netherlands	CSF	C	11	11	a1	nd
991379	1999	The Netherlands	CSF	C	11	11	a1	nd
991930	1999	The Netherlands	CSF	C	11	11	a1	nd
991932	1999	The Netherlands	CSF	C	11	11	a1	nd
992062	1999	The Netherlands	CSF	C	11	11	a1	nd
992076	1999	The Netherlands	CSF	C	11	11	a1	nd
2000151	2000	The Netherlands	CSF	C	11	11	a1	nd
2000171	2000	The Netherlands	CSF	C	11	11	a1,b,c,d	a, b
2000321	2000	The Netherlands	CSF	C	11	11	a1	nd
2000345	2000	The Netherlands	CSF	C	11	11	a1	nd
2000449	2000	The Netherlands	CSF	C	11	11	a1	nd
2000739	2000	The Netherlands	CSF	C	11	11	a1,b,c,d	a
2000779	2000	The Netherlands	CSF	C	11	11	a1	nd
2000780	2000	The Netherlands	CSF	W135	11	11	a1	nd
2000811	2000	The Netherlands	CSF	W135	11	11	a1	nd
2000844	2000	The Netherlands	Eye	W135	11	11	a1	nd
2000907	2000	The Netherlands	Blood	W135	11	11	a1,b,c,d	a
2000948	2000	The Netherlands	CSF	C	11	11	a1	nd
2000974	2000	The Netherlands	CSF	C	11	11	a1	nd
2001044	2000	The Netherlands	Blood	C	11	11	c[1]	a
2001188	2000	The Netherlands	Blood	C	11	11	a1	nd
2001212	2000	The Netherlands	CSF	C	11	11	a1	nd
2001215	2000	The Netherlands	Blood	C	11	11	a1	nd
2001271	2000	The Netherlands	Blood	C	11	11	a1	nd
2001329	2000	The Netherlands	CSF	C	2704	11	a1	nd
2001330	2000	The Netherlands	CSF	C	1055	11	a1	nd
2001428	2000	The Netherlands	Blood	C	11	11	a1,b,c,d	a
2001477	2000	The Netherlands	CSF	C	11	11	a1	nd
2001540	2000	The Netherlands	CSF	C	11	11	a1	nd
2001622	2000	The Netherlands	Blood	C	11	11	a1	nd
2001623	2000	The Netherlands	Blood	C	11	11	a1	nd
2001633	2000	The Netherlands	CSF	C	11	11	a1,b,c,d	b
2001718	2000	The Netherlands	CSF	C	11	11	a1	nd
2001730	2000	The Netherlands	CSF	C	11	11	a1	nd
2001848	2000	The Netherlands	Blood	C	11	11	a1,b,c,d	a
2001861	2000	The Netherlands	CSF	C	11	11	a1	nd
2001953	2000	The Netherlands	CSF	C	11	11	a1	nd
2002038	2000	The Netherlands	CSF	C	51	11	a1	nd
2002173	2000	The Netherlands	CSF	C	3456	11	a1,b,c,d	
2002188	2000	The Netherlands	CSF	W135	11	11	a1	nd
2010131	2001	The Netherlands	CSF	C	11	11	a1	nd
2010178	2001	The Netherlands	CSF	C	11	11	a1	nd
2010250	2001	The Netherlands	Blood	C	11	11	a1,b,c,d	nd
2010291	2001	The Netherlands	Blood	C	11	11	a1	nd
2010351	2001	The Netherlands	Blood	W135	247	11	a1	nd
2010394	2001	The Netherlands	CSF	C	11	11	a1	nd
2010443	2001	The Netherlands	Blood	C	11	11	a1	nd
2010445	2001	The Netherlands	Blood	C	11	11	a1,b,c,d	a

2010478	2001	The Netherlands	Blood	C	11	11	a1	nd
2010513	2001	The Netherlands	CSF	C	11	11	a1	nd
2010563	2001	The Netherlands	CSF	C	11	11	a1	nd
2010664	2001	The Netherlands	CSF	C	11	11	a1	nd
2010699	2001	The Netherlands	CSF	C	11	11	a1	nd
2010760	2001	The Netherlands	CSF	C	11	11	a1,b,c,d	a
2010816	2001	The Netherlands	CSF	C	11	11	a1	nd
2010818	2001	The Netherlands	Blood	C	11	11	a1	nd
2010878	2001	The Netherlands	CSF	C	11	11	a1	nd
2010905	2001	The Netherlands	Blood	C	11	11	a1	nd
2010938	2001	The Netherlands	Blood	C	11	11	a1	nd
2010939	2001	The Netherlands	CSF	C	11	11	a1	nd
2010962	2001	The Netherlands	CSF	C	11	11	a1	nd
2010980	2001	The Netherlands	Blood	C	11	11	a1	nd
2011000	2001	The Netherlands	CSF	C	11	11	a1,b,c,d	b
2011043	2001	The Netherlands	CSF	C	11	11	a1	nd
2011061	2001	The Netherlands	Blood	C	11	11	a1	nd
2011150	2001	The Netherlands	CSF	C	11	11	a1	nd
2011186	2001	The Netherlands	Blood	C	11	11	a1,b,c,d	a
2011212	2001	The Netherlands	CSF	C	11	11	a1	nd
2011215	2001	The Netherlands	CSF	C	11	11	a1	nd
2011255	2001	The Netherlands	Blood	C	11	11	a1,b,c,d	b
2011328	2001	The Netherlands	Blood	C	11	11	a1	nd
2011333	2001	The Netherlands	Blood	B	11	11	a1,b,c,d	a, b
2011334	2001	The Netherlands	CSF	C	11	11	a1	nd
2011335	2001	The Netherlands	CSF	C	11	11	a1	nd
2011358	2001	The Netherlands	CSF	C	11	11	a1	nd
2011371	2001	The Netherlands	CSF	C	11	11	a1	nd
2011471	2001	The Netherlands	CSF	C	11	11	a1	nd
2011528	2001	The Netherlands	CSF	C	2704	11	a1	nd
2011545	2001	The Netherlands	Blood	C	11	11	a1	nd
2011576	2001	The Netherlands	Blood	C	2704	11	a1,b,c,d	a
2011603	2001	The Netherlands	CSF	C	11	11	a1	nd
2011674	2001	The Netherlands	CSF	C	11	11	a1	nd
2011692	2001	The Netherlands	CSF	C	11	11	a1	nd
2011709	2001	The Netherlands	Blood	C	11	11	a1	nd
2011728	2001	The Netherlands	CSF	C	4283	11	a1	nd
2011748	2001	The Netherlands	CSF	C	11	11	a1	nd
2011780	2001	The Netherlands	CSF	C	11	11	a1,b,c,d	
2011784	2001	The Netherlands	Blood	C	11	11	a1	nd
2011785	2001	The Netherlands	Blood	C	11	11	a1	nd
2011791	2001	The Netherlands	Blood	C	11	11	a1,b,c,d	a
2011792	2001	The Netherlands	Blood	C	11	11	a1	nd
2011793	2001	The Netherlands	CSF	C	11	11	a1	nd
2011801	2001	The Netherlands	Blood	C	11	11	a1	nd
2011805	2001	The Netherlands	CSF	C	11	11	a1	nd
2011814	2001	The Netherlands	Blood	C	11	11	a1	nd
2011815	2001	The Netherlands	Blood	C	11	11	a1,b,c,d	a, b
2011823	2001	The Netherlands	Blood	C	11	11	a1	nd
2011831	2001	The Netherlands	CSF	C	11	11	a1	nd
2011832	2001	The Netherlands	CSF	C	11	11	a1	nd
2011852	2001	The Netherlands	CSF	C	11	11	a1	nd
2011872	2001	The Netherlands	Blood	C	247	11	a1	nd

2011929	2001	The Netherlands	CSF	C	11	11	a1	nd
2011940	2001	The Netherlands	CSF	W135	11	11	a1	nd
2011975	2001	The Netherlands	Blood	C	247	11	a1	nd
2011978	2001	The Netherlands	CSF	C	247	11	a1	nd
2011980	2001	The Netherlands	CSF	C	11	11	a1	nd
2012005	2001	The Netherlands	CSF	C	11	11	a1	nd
2012024	2001	The Netherlands	Blood	C	11	11	a1	nd
2012062	2001	The Netherlands	CSF	C	11	11	a1	nd
2012099	2001	The Netherlands	Blood	C	11	11	a1	nd
2012140	2001	The Netherlands	Blood	C	11	11	a1	nd
2012199	2001	The Netherlands	Blood	C	11	11	a1,b,c,d	a
2012210	2001	The Netherlands	Blood	C	11	11	a1	nd
2012278	2001	The Netherlands	Blood	C	11	11	a1	nd
2012280	2001	The Netherlands	CSF	C	11	11	a1	nd
2012300	2001	The Netherlands	Blood	C	11	11	a1	nd
2012322	2001	The Netherlands	CSF	C	11	11	a1,b,c,d	b
2012341	2001	The Netherlands	Blood	C	11	11	a1	nd
2012376	2001	The Netherlands	Blood	C	11	11	a1	nd
2012402	2001	The Netherlands	Blood	C	2706	11	a1	nd
2012427	2001	The Netherlands	Blood	C	11	11	a1	nd
2012431	2001	The Netherlands	CSF	C	11	11	a1,b,c,d	a
2012448	2001	The Netherlands	Blood	C	11	11	a1	nd
2012526	2001	The Netherlands	CSF	C	11	11	a1	nd
2012532	2001	The Netherlands	Blood	C	11	11	a1	nd
2012588	2001	The Netherlands	Blood	C	11	11	a1	nd
2012598	2001	The Netherlands	CSF	C	11	11	a1	nd
2012602	2001	The Netherlands	CSF	C	11	11	a1	nd
2012620	2001	The Netherlands	CSF	C	11	11	a1	nd
2012622	2001	The Netherlands	Blood	C	11	11	a1	nd
2012655	2001	The Netherlands	CSF	C	11	11	a1	nd
2012671	2001	The Netherlands	CSF	W135	11	11	a1	nd
2012673	2001	The Netherlands	CSF	C	2709	11	a1,b,c,d	a, b
2012717	2001	The Netherlands	CSF	C	11	11	a1	nd
2020004	2002	The Netherlands	CSF	C	11	11	a1	nd
2020017	2002	The Netherlands	CSF	C	11	11	a1	nd
2020041	2002	The Netherlands	Blood	C	11	11	c[1]	
2020047	2002	The Netherlands	CSF	C	11	11	a1	nd
2020130	2002	The Netherlands	CSF	C	11	11	a1	nd
2020145	2002	The Netherlands	CSF	C	11	11	a1	nd
2020148	2002	The Netherlands	Blood	C	11	11	a1	nd
2020212	2002	The Netherlands	CSF	C	11	11	a1	nd
2020218	2002	The Netherlands	Blood	C	11	11	a1,b,c,d	a
2020227	2002	The Netherlands	CSF	C	1254	11	a1	nd
2020236	2002	The Netherlands	CSF	C	11	11	a1	nd
2020258	2002	The Netherlands	CSF	C	11	11	a1,b,c,d	b
2020279	2002	The Netherlands	Blood	C	11	11	a1	nd
2020322	2002	The Netherlands	Blood	C	11	11	a1	nd
2020328	2002	The Netherlands	CSF	C	11	11	a1	nd
2020349	2002	The Netherlands	Blood	C	11	11	a1	nd
2020370	2002	The Netherlands	Blood	C	11	11	a1,b,c,d	a, b
2020397	2002	The Netherlands	CSF	C	3035	11	a1	nd
2020435	2002	The Netherlands	CSF	C	3298	11	a1	nd
2020448	2002	The Netherlands	CSF	C	11	11	a1	nd

2020503	2002	The Netherlands	CSF	C	11	11	a1	nd
2020515	2002	The Netherlands	CSF	C	11	11	a1	nd
2020530	2002	The Netherlands	Blood	C	11	11	a1	nd
2020533	2002	The Netherlands	Blood	C	11	11	a1	nd
2020540	2002	The Netherlands	CSF	C	11	11	a1	nd
2020561	2002	The Netherlands	CSF	C	11	11	a1	nd
2020570	2002	The Netherlands	Blood	C	11	11	a1,b,c,d	a
2020602	2002	The Netherlands	Blood	C	11	11	a1	nd
2020629	2002	The Netherlands	Blood	C	11	11	a1	nd
2020637	2002	The Netherlands	Blood	C	3726	11	a1	nd
2020656	2002	The Netherlands	CSF	C	11	11	a1	nd
2020680	2002	The Netherlands	Blood	C	11	11	a1	nd
2020690	2002	The Netherlands	Blood	C	11	11	a1,b,c,d	b
2020693	2002	The Netherlands	CSF	C	11	11	a1	nd
2020712	2002	The Netherlands	CSF	C	11	11	a1	nd
2020730	2002	The Netherlands	CSF	C	11	11	a1	nd
2020748	2002	The Netherlands	CSF	C	11	11	a1	nd
2020844	2002	The Netherlands	Blood	C	11	11	a1	nd
2020875	2002	The Netherlands	CSF	C	11	11	a1	nd
2020878	2002	The Netherlands	Blood	C	11	11	a1	nd
2020910	2002	The Netherlands	Blood	C	11	11	a1	nd
2020955	2002	The Netherlands	Blood	C	11	11	a1,b,c,d	
2020968	2002	The Netherlands	Blood	C	11	11	a1	nd
2020982	2002	The Netherlands	CSF	C	3732	11	a1	nd
2021027	2002	The Netherlands	CSF	C	11	11	a1	nd
2021181	2002	The Netherlands	Blood	C	11	11	a1,b,c,d	a, b
2021264	2002	The Netherlands	Blood	C	11	11	a1	nd
2021267	2002	The Netherlands	CSF	C	11	11	a1	nd
2021306	2002	The Netherlands	Blood	C	11	11	a1	nd
2021322	2002	The Netherlands	Blood	C	11	11	a1	nd
2021335	2002	The Netherlands	CSF	C	11	11	a1,b,c,d	
2021352	2002	The Netherlands	Blood	C	11	11	a1	nd
2021468	2002	The Netherlands	CSF	C	11	11	a1	nd
2021481	2002	The Netherlands	Blood	C	11	11	a1	nd
2021708	2002	The Netherlands	Blood	C	2704	11	a1	nd
2021728	2002	The Netherlands	CSF	C	11	11	a1	nd
2021740	2002	The Netherlands	Blood	C	11	11	a1	nd
2021775	2002	The Netherlands	CSF	B	11	11	a1	nd
2021819	2002	The Netherlands	CSF	C	3456	11	a1	nd
2021907	2002	The Netherlands	CSF	C	11	11	a1,b,c,d	a, b
2021940	2002	The Netherlands	CSF	C	11	11	a1	nd
2022024	2002	The Netherlands	CSF	C	11	11	a1	nd
2022505	2002	The Netherlands	CSF	C	11	11	a1	nd
2030100	2003	The Netherlands	CSF	C	11	11	a1	nd
2030399	2003	The Netherlands	Blood	C	11	11	a1,b,c,d	a, b
2030488	2003	The Netherlands	CSF	C	11	11	a1	nd
2030689	2003	The Netherlands	Blood	C	11	11	a1	nd
2031022	2003	The Netherlands	CSF	C	11	11	a1	nd
2031338	2003	The Netherlands	CSF	C	11	11	a1	nd
2031356	2003	The Netherlands	CSF	C	11	11	a1	nd
2031499	2003	The Netherlands	Blood	C	11	11	a1	nd
2031503	2003	The Netherlands	Blood	C	11	11	a1	nd
2031588	2003	The Netherlands	Blood	C	11	11	a1,b,c,d	a, b

2032645	2003	The Netherlands	Blood	C	11	11	a1	nd
2032737	2003	The Netherlands	Blood	C	11	11	a1	nd
2040215	2004	The Netherlands	Blood	C	11	11	a1,b,c,d	a, b
2040237	2004	The Netherlands	CSF	C	11	11	a1	nd
2041114	2004	The Netherlands	Blood	C	1026	11	a1	nd
2041350	2004	The Netherlands	Blood	B	5412	11	a1	nd
2050123	2005	The Netherlands	Blood	C	11	11	a1	nd
2051166	2005	The Netherlands	CSF	W135	11	11	a1,b,c,d	
2051624	2005	The Netherlands	CSF	B	11	11	a1	nd
2060153	2006	The Netherlands	Blood	C	11	11	a1	nd
2061255	2006	The Netherlands	Blood	C	11	11	a1	nd
2070078	2007	The Netherlands	CSF	C	3298	11	a1	nd
2071345	2007	The Netherlands	CSF	C	11	11	a1,b,c,d	a
2071415	2007	The Netherlands	CSF	W135	11	11	a1	nd
2071442	2007	The Netherlands	CSF	C	11	11	a1	nd
2071467	2007	The Netherlands	CSF	W135	11	11	a1	nd
2071886	2007	The Netherlands	Blood	C	11	11	a1,b,c,d	
2080098	2008	The Netherlands	CSF	C	211	11	a1	nd
2080150	2008	The Netherlands	Blood	C	6872	11	a1	nd
2080184	2008	The Netherlands	CSF	C	11	11	a1	nd
α122	1999/00	Germany	NSF	W135	11	11	a1,a2,b	b
α223	1999/00	Germany	NSF	C	11	11	a1,a2,b	a, b
α306	1999/00	Germany	NSF	W135	11	11	a1,a2,b	
α324	1999/00	Germany	NSF	C	11	11	a1,a2,b	a
α737	1999/00	Germany	NSF	C	11	11	a1,a2,b	b
α738	1999/00	Germany	NSF	C	11	11	a1,a2,b	
2996[#]	1975	United kingdom	invasive	B	540	8	a1,a2,b	nd
149	1961	The Netherlands	CSF	B	6008	8	a1	nd
189	1962	The Netherlands	CSF	B	8	8	a1	nd
227	1962	The Netherlands	CSF	B	8	8	a1	nd
245	1962	The Netherlands	CSF	B	6884	8	a1	nd
424	1964	The Netherlands	CSF	B	8	8	a1	nd
1679	1970	The Netherlands	CSF	B	487	8	a1	nd
1685	1970	The Netherlands	CSF	B	8	8	a1	nd
1692	1970	The Netherlands	CSF	B	8	8	a1	nd
1707	1970	The Netherlands	Blood	B	8	8	a1	nd
1717	1970	The Netherlands	CSF	B	8	8	a1	nd
1726	1970	The Netherlands	CSF	B	8	8	a1	nd
1744	1970	The Netherlands	CSF	B	4860	8	a1	nd
1767	1970	The Netherlands	CSF	B	2174	8	a1	nd
1768	1970	The Netherlands	CSF	B	8	8	a1	nd
1789	1970	The Netherlands	CSF	B	8	8	a1	nd
1796	1970	The Netherlands	CSF	B	8	8	a1	nd
1828	1970	The Netherlands	CSF	B	8	8	a1	nd
1838	1970	The Netherlands	CSF	B	8	8	a1	nd
1914	1970	The Netherlands	CSF	B	8	8	a1	nd
1926	1970	The Netherlands	CSF	B	8	8	a1	nd
1949	1970	The Netherlands	CSF	B	2174	8	a1	nd
800001	1980	The Netherlands	CSF	B	6853	8	a1,b,d	nd
800062	1980	The Netherlands	Blood	B	8	8	a1,b,d	nd
800083	1980	The Netherlands	CSF	B	8	8	a1,b,d	nd
800094	1980	The Netherlands	CSF	B	8	8	a1	nd
800236	1980	The Netherlands	CSF	B	9	8	a1	nd

800254	1980	The Netherlands	CSF	B	6856	8	a1	nd
800297	1980	The Netherlands	CSF	B	2174	8	a1	nd
800426	1980	The Netherlands	CSF	B	8	8	a1	nd
800624	1980	The Netherlands	CSF	B	8	8	a1	nd
800702	1980	The Netherlands	CSF	B	4611	8	a1	nd
801356	1980	The Netherlands	Blood	B	8	8	a1	nd
850223	1985	The Netherlands	CSF	B	9	8	a1	nd
850272	1985	The Netherlands	Blood	B	335	8	a1	nd
850387	1985	The Netherlands	CSF	B	7102	8	a1	nd
850519	1985	The Netherlands	CSF	B	8	8	a1	nd
851347	1985	The Netherlands	CSF	B	9	8	a1	nd
902388	1990	The Netherlands	CSF	B	153	8	a1	nd
982084	1998	The Netherlands	CSF	C	66	8	a1	nd
991210	1999	The Netherlands	Blood	C	2680	8	a1	nd
2000221	2000	The Netherlands	Blood	C	66	8	a1	nd
2000235	2000	The Netherlands	CSF	C	2680	8	a1	nd
2000640	2000	The Netherlands	CSF	C	66	8	a1	nd
2000863	2000	The Netherlands	CSF	C	2680	8	a1	nd
2000966	2000	The Netherlands	CSF	C	66	8	a1	nd
2001031	2000	The Netherlands	Blood	C	2680	8	a1	nd
2001173	2000	The Netherlands	CSF	C	4428	8	a1	nd
2001346	2000	The Netherlands	CSF	C	2680	8	a1	nd
2001359	2000	The Netherlands	CSF	B	4429	8	a1	nd
2001509	2000	The Netherlands	CSF	C	2699	8	a1	nd
2001649	2000	The Netherlands	CSF	C	66	8	a1	nd
2001821	2000	The Netherlands	CSF	C	2680	8	a1	nd
2002111	2000	The Netherlands	Blood	C	2680	8	a1	nd
2010115	2001	The Netherlands	CSF	C	2699	8	a1	nd
2010221	2001	The Netherlands	CSF	C	2680	8	a1	nd
2010383	2001	The Netherlands	CSF	C	2680	8	a1	nd
2010403	2001	The Netherlands	CSF	C	2699	8	a1	nd
2010511	2001	The Netherlands	CSF	C	2699	8	a1	nd
2010745	2001	The Netherlands	CSF	C	8	8	a1	nd
2010937	2001	The Netherlands	Blood	C	2680	8	a1	nd
2011060	2001	The Netherlands	CSF	C	2699	8	a1	nd
2011437	2001	The Netherlands	Blood	C	2680	8	a1	nd
2011578	2001	The Netherlands	Blood	C	66	8	a1	nd
2011726	2001	The Netherlands	CSF	C	1372	8	a1	nd
2011899	2001	The Netherlands	Blood	C	2680	8	a1	nd
2012063	2001	The Netherlands	Blood	C	2680	8	a1	nd
2012403	2001	The Netherlands	Throat	C	2680	8	a1	nd
2012552	2001	The Netherlands	CSF	C	2680	8	a1	nd
2012637	2001	The Netherlands	Blood	C	2680	8	a1	nd
2012653	2001	The Netherlands	CSF	C	2680	8	a1	nd
2012711	2001	The Netherlands	Blood	C	8	8	a1	nd
2020151	2002	The Netherlands	CSF	C	3486	8	a1,b,d	nd
2020745	2002	The Netherlands	CSF	C	2680	8	a1,b,d	nd
2020907	2002	The Netherlands	Blood	C	1372	8	a1	nd
2020933	2002	The Netherlands	CSF	C	2680	8	a1	nd
2020945	2002	The Netherlands	CSF	C	2680	8	a1	nd
2020994	2002	The Netherlands	CSF	C	2680	8	a1	nd
2021497	2002	The Netherlands	CSF	C	2680	8	a1	nd
2021581	2002	The Netherlands	Blood	C	3522	8	a1	nd

2021671	2002	The Netherlands	Blood	C	1372	8	a1,b,d	nd
2040210	2004	The Netherlands	CSF	C	8	8	a1,b,d	nd
2040355	2004	The Netherlands	Blood	B	153	8	a1,b,d	nd
2040362	2004	The Netherlands	CSF	C	8	8	a1	nd
2041497	2004	The Netherlands	CSF	C	1372	8	a1	nd
2060591	2006	The Netherlands	Blood	C	66	8	a1	nd
2061599	2006	The Netherlands	CSF	C	8	8	a1	nd
2071065	2007	The Netherlands	CSF	C	6859	8	a1	nd
2071129	2007	The Netherlands	Blood	C	1372	8	a1	nd
2071211	2007	The Netherlands	CSF	C	6861	8	a1	nd
2071237	2007	The Netherlands	Blood	C	1372	8	a1	nd
2080039	2008	The Netherlands	Blood	C	1372	8	a1	nd
2080070	2008	The Netherlands	blood / CSF	B	32	32	f^2,p,e^2	nd
2071282	2007	The Netherlands	CSF	B	259	32	f,p,e^2	nd
2092035	2009	The Netherlands	blood / CSF	B	34	32	f,p,e,v	nd
2090919	2009	The Netherlands	blood	B	34	32	f,p,e,v	nd
2090516	2009	The Netherlands	blood	B	32	32	f,e,v,q	nd
2090354	2009	The Netherlands	CSF	B	3720	32	f,p,e,v	nd
2090110	2009	The Netherlands	blood / CSF	B	33	32	f,p,e,v	nd
2090092	2009	The Netherlands	blood	B	34	32	f,p,e,v	nd
2082287	2008	The Netherlands	CSF	B	33	32	f,e,v,q	nd
2082151	2008	The Netherlands	CSF	B	3720	32	f,p,e,v	nd
2081656	2008	The Netherlands	blood / CSF	B	32	32	f,e,v,q	nd
2081056	2008	The Netherlands	blood / CSF	B	34	32	f,p,e,v	nd
2080724	2008	The Netherlands	blood / CSF	B	7884	32	f,e,v,q	nd
2080676	2008	The Netherlands	CSF	B	32	32	f,p,e,v	nd
2080654	2008	The Netherlands	CSF	B	34	32	f,p,e,v	nd
2080564	2008	The Netherlands	joint	B	34	32	f,p,e,v	nd
2080448	2008	The Netherlands	blood	B	34	32	f,p,e,v	nd
2080151	2008	The Netherlands	blood	B	32	32	f,p,e	nd
2071909	2007	The Netherlands	blood	B	3720	32	f,p,e,v	nd
2071749	2007	The Netherlands	blood	B	34	32	f,p,e,v	nd
2071499	2007	The Netherlands	blood / CSF	B	5138	32	f,p,e,q	nd
2071291	2007	The Netherlands	blood	B	3720	32	f,p,e,v	nd
2071066	2007	The Netherlands	blood	B	34	32	f,p,e	nd
2070973	2007	The Netherlands	blood	B	34	32	f,p,e,v	nd
2070947	2007	The Netherlands	CSF	B	6299	32	f,e,v^2,q	nd
2070929	2007	The Netherlands	blood / CSF	B	32	32	f,e,v,q	nd
2070861	2007	The Netherlands	blood / CSF	B	32	32	f,e,v,q	nd
2070755	2007	The Netherlands	blood	B	4949	32	f,p,e,v	nd
2070582	2007	The Netherlands	CSF	B	33	32	f,e,v,q	nd
2070472	2007	The Netherlands	throat	B	6292	32	f,p,e,q	nd
2070206	2007	The Netherlands	blood / CSF	B	3720	32	f,p,e,v	nd

2070118	2007	The Netherlands	blood / CSF	B	34	32	f,p,e,v	nd
2070093	2007	The Netherlands	CSF	B	3720	32	f,p,e,v	nd
2070077	2007	The Netherlands	blood	B	34	32	f,p,e,	nd
2061546	2006	The Netherlands	blood	B	34	32	f,p,e,v	nd
2061481	2006	The Netherlands	blood	B	32	32	f,p,e	nd
2061322	2006	The Netherlands	blood	B	32	32	f,e,v,q	nd
2061285	2006	The Netherlands	CSF	B	3720	32	f,p,e,v	nd
2061227	2006	The Netherlands	CSF	B	5955	32	f,e,q	nd
2061192	2006	The Netherlands	blood	B	34	32	f,p,e,v	nd
2061147	2006	The Netherlands	CSF	B	3720	32	f,p,e,v	nd
2061119	2006	The Netherlands	blood / CSF	B	33	32	f,p,e,v	nd
2060978	2006	The Netherlands	blood	B	34	32	f,p,e,v	nd
2060939	2006	The Netherlands	blood	B	34	32	f,p,e,v	nd
2060640	2006	The Netherlands	blood	B	3720	32	f,p,e,v	nd
2060602	2006	The Netherlands	CSF	B	33	32	f,p,e	nd
2060489	2006	The Netherlands	blood	B	33	32	f,p,e	nd
2081107	2008	The Netherlands	blood	B	34	32	f,q,r,s, d[2],b	nd
2061646	2006	The Netherlands	CSF	B	32	32	f,q	nd
2061468	2006	The Netherlands	blood	B	34	32	f,q,r,s,t	nd
α153[#]	1999/00	Germany	NSF	29E	60	60	r4	nd
α14[#]	1999/00	Germany	NSF	cnI	53	53	r3	nd

[a] #, *Neisseria* reference strains.
[b] Invasive, disease isolate without specified origin; CSF, cerebrospinal fluid; NSF, carrier isolates from nasopharynx.
[c] DNA fragments amplified by PCR as specified in Figures S4 and S6 in Additional file 1 and Table S3 in Additional file 3. Superscripts 1 and 2 indicate that the amplicons were smaller and larger, respectively, than expected. Note that all cc11 and cc8 isolates were initially tested for amplification of fragment *a1,* while only some of them were subsequently tested for other fragments. Results for fragments j-n are not shown. Fragments *e* and *v* indicate positive tests for the presence of the *pilE* locus and the same hypervariable region of *pilE* as in MC58, respectively. Fragments *q*, *r* and *s* were tested in cc32 isolates negative for fragment *p*. The last two strains listed were not extensively evaluated but used for specific purposes only; only fragments obtained for those assays are indicated.
[d] Positive reactions in Western blots with antiserum directed against NalP (a) and a mixture of monoclonal antibodies recognizing OpaA, OpaB, OpaD and OpaJ (b) are indicated. nd, no reactions determined.
