# Supplementary material for: Domain exchange at the 3’ end of the gene encoding the fratricide meningococcal two-partner secretion protein A
Source: BMC Genomics. 2013 Sep 14;14:622. doi: 10.1186/1471-2164-14-622 (PMC3848433; doi:10.1186/1471-2164-14-622)
Supplement: Additional file 3: Table S3 — listing the PCR primers used in this study. [file 1471-2164-14-622-S3.pdf]

**Table S3**. Primers used in this study

| Fragment[a] | sequence (5'->3') | locus tag[b] | amplicon size[c] | remarks[d] |
|---|---|---|---|---|
| *a1* | CAGGAAATTTGATTACTACATACCCAGTA | NMC0444 | 1098 | |
| | GGAGGCTAAAGCTGCGGTTTGCATGGCAGCTGC | NMC0446 | | |
| *a2* | CAGGAAATTTGATTACTACATACCCAGTA | NMC0444 | 2651 | |
| | GGATCCTTATTGGAATTTAAGCTCCAATTTAGTA | NMC0446 | | |
| *b* | CCATTGACATCAGCGGTGTA | NMC0444 | 2390 | |
| | AGCCAGCAGATACCGGTTGTCCCGTTTGTCTCA | NMC0444 | | |
| *c* | CCATTGACATCAGCGGTGTA | NMC0444 | 5342 | |
| | GGATCCTTATTGGAATTTAAGCTCCAATTTAGTA | NMC0446 | | |
| *d* | ACTGCAGAAAAGAATCTGGA | NMC0444 | 2280 | |
| | CCACCCAGTGTGGTTTCAA | NMC0444 | | |
| *e* | GCCAAGGCCGATGTCAATGC | NMA0688 | 1596 | |
| | TGCCGCCTGCCTGAAGCGT | NMA0688 | | |
| *f* | GAAATTGGCAAGCCCACCTA | NMB0497 | 2415 | |
| | AGCCAGCAGATACCGGTTGTCCCGTTTGTCTCA | NMB0497 | | |

| | | | | |
|---|---|---|---|---|
| *g* | GAAATTGGCAAGCCCACCTA | NMA0688 | 5210 | |
| | GCAGTCATTTCGTTATCAA | NMA0690 | | |
| *h1* | GAGGATCCGAATTGCGCCACCGTGCTTA | NMC0443 | | BamHI |
| | CTCTGCAGGTTTGACGGTCTGAGGATAAGGAAAGA | NMC0443 / NMC0444 | | PstI |
| *h2* | AAAGGCGCCATTTACAAAGGATTTTACCG | NMC0447 | | KasI |
| | AAAAAACACAACGTGGGGACACATTAAAAACAGAAT | NMC0448 | | DraIII |
| *i* | AAACTGCAGTGATTTTCTACTTAAATCAAGCTACCTTAA | NMC0458 | | PstI |
| | AAACATATGTTATTTTATCTTCTGAAAGAAAATTATTTTTTT | NMC0459 | | NdeI |
| *j* | TGGACATTAGATTTGCAGAATCC | NMC0446 | 2222 | |
| | AAAGCCGGCTATGCAGAAATGATTTTATT | NMC0448 | | |
| *k* | AATGATTCATCCGTATATCT | NMC0448 | 2853 | |
| | GGATCCCTATTTTCCTGTAAAAATATTTAATTG | NMC0450 | | |
| *l* | CTCTCCTCCTAGAGGAACAT | NMC0450 | 2817 | |
| | TTGTCCACGGCTCTCTAGGATTCCTCATA | NMC0452 | | |
| *m* | GTACACCTGCTTCGCTAAG | NMC0452 | 1862 | |
| | AGAGTCAAATAACTGCTCGGTCATTTTAC | NMC0456 | | |
| *n* | TATGATTTACAATACTCTGCTGAT | NMC0456 | 2073 | |
| | TAATAGCAAGAGTCTGAAGCAAA | NMC0459 | | |

| | | | |
|---|---|---|---|
| *p* | GAAATTGGCAAGCCCACCTA | NMB1779 | 2774 |
| | GGATCCTTATTGGAATTTAAGCTCCAATTTAGTA | NMB1779 | |
| *q* | GCTGCCTGTTATCCTGCAAACAGA | Upstream NMB1780 | 3303 |
| | CAAAGGATCCTAAGCATTTAATACCGTGGTAGCTGGGCGA | NMB1779 | |
| *r* | GCCCTCGCGTTTGGCATTTTCAGCAA | NMB0493 | 1875 |
| | CCGAGTACACGGTGTTTCATCTTCACT | NMB0496 | |
| *r1* | CATATGGCTGCTCCTAAAGAAACAATAAAC | NMC0446 | |
| | AGCGTCGACTTCAGACGGCCACGTTGTGTC | *kan* cassette | |
| *r2* | CCATTGACATCAGCGGTGTA | NMO_0398 | |
| | AGCGTCGACTTCAGACGGCCACGTTGTGTC | *kan* cassette | |
| *r3* | CCATTGACATCAGCGGTGTA | NMO_0398 | 2623 |
| | AGAGTCAAATAACTGCTCGGTCATTTTAC | NMO_0398 | |
| *r4* | GAAATTGGCAAGCCCACCTA | NME_0860 | 2722 |
| | GGATCCTTATTGGAATTTAAGCTCCAATTTAGTA | NME_0860 | |
| *s* | CCACAGCCCCACGTTTGCGGTT | NMB1771 | 1710 |
| | CAGGATCCATTTTCACCTAAACTATGCACGCTAGA | NMB1768 | |
| *t* | GAAATTGGCAAGCCCACCTA | NMB1779 / NMB0497 | |
| | CAAAGACTACCACCAACA | NMB0511 | |

| | | | | |
|---|---|---|---|---|
| *v* | CGCGCGCATATGAGTAACTTTGAAAAAAAATATA | NMC0445 | 489 | NdeI |
| | GCGCGCGACGTCTTATTTAAATTCATCACGTTTCA | NMC0445 | | AatII |

[a] Letters correspond to the PCR fragments indicated in Figures S4 and S6 in Additional file 1and Table S2 in Additional file 2.
[b] Locus tags are from the genome sequences of FAM18, Z2491, MC58, α14 and α153.
[c] Amplicon size given is based on the available genome sequences of the reference strains.
[d] Restriction enzymes used for cloning. The restriction sites are underlined in the primer sequences.
